# Contributions of the basolateral amygdala and nucleus accumbens to sustaining not just initiating cognitive effort

**DOI:** 10.1073/pnas.2601231123

**Published:** 2026-05-06

**Authors:** Matthew L. Dixon, Elizabeth Blevins, Carol S. Dweck, Kai Görgen, Brian Knutson

**Affiliations:** ^a^ Department of Psychology, Stanford University, Stanford, CA 94305; ^b^ Bernstein Center for Computational Neuroscience Berlin and Berlin Center for Advanced Neuroimaging, Charité Universitätsmedizin Berlin, corporate member of the Freie Universität Berlin and Humboldt Universität zu Berlin, Berlin 10115, Germany

**Keywords:** amygdala, nucleus accumbens, effort, cognitive control, reward

## Abstract

People must often sustain cognitive effort to achieve important goals. Although current models recognize that subcortical valuation regions are important for initiating such effort, they have not seen them as playing meaningful roles in sustaining effort throughout task performance. Here, we demonstrate that core subcortical valuation regions—the basolateral amygdala (BLA) and nucleus accumbens (NAcc)—contribute to sustaining cognitive effort on a challenging working memory task. First, convergent univariate and multivariate analyses revealed that the BLA and NAcc represented incentive value, cognitive effort demands, or both, during every task period (encoding, maintenance, and probe/response). Second, trial-to-trial fluctuations in BLA/NAcc multivariate value coding predicted both the strength of frontoparietal cortical engagement and behavioral performance. Third, the BLA and NAcc functionally interacted with frontoparietal regions throughout the task, suggesting that they work in a coordinated manner. These findings reveal a continuous and dynamic role for subcortical valuation regions in sustaining cognitive effort.

## Significance

How does the brain sustain effortful cognitive activity? Might subcortical valuation regions play a larger role than they are usually given credit for? Using neuroimaging with humans, we show that the amygdala and the nucleus accumbens work together with frontoparietal cortical regions during a difficult working memory task. Moreover, this subcortical activity predicted both the degree to which frontoparietal regions were engaged on each trial and the level of cognitive performance individuals displayed. These findings challenge the idea that subcortical valuation regions are primarily limited to stimulus-driven reward processing and instead suggest that they play a sustained role in supporting executive functions.

A fundamental puzzle is why individuals sometimes persist at difficult cognitive tasks, yet at other times have difficulty sustaining cognitive effort, despite having the necessary abilities (1–4). Early perspectives suggested that subcortical “emotion regions” such as the amygdala and nucleus accumbens (NAcc) were either minimally involved in sustaining cognitive effort, or were detrimental to it, contributing interference that could disrupt attention and working memory (5–7).

However, the now-dominant account of cognitive effort in the field (1, 2, 8–15)—concisely summarized in the expected value of control (EVC) framework—emphasizes a role for valuation. Specifically, individuals choose whether to engage effortful cognitive control processes such as working memory by initially weighing expected benefits against the costs. Consistent with such an account, recent research has established a useful, albeit limited role for valuation regions including the amygdala and NAcc, showing that their activity can reflect anticipated costs and benefits when incentive cues are presented before exertion of cognitive effort (16–20). However, the potential contribution of these regions during the performance of demanding tasks—when cognitive effort needs to be sustained—has yet to be demonstrated. In this paper, “sustaining cognitive effort” refers to the moment-to-moment support of effortful cognitive control processes throughout task execution during each trial, which can be clearly distinguished from an initial decision to initiate effort.

Here, we propose that these core subcortical valuation regions contribute more to the ability to sustain cognitive effort than commonly assumed (21). Specifically, amygdala (22–26) and NAcc (27–30) value-coding activity might persist throughout task performance, signaling moment-to-moment variation in the value of exerting cognitive effort, and thereby allowing individuals to persist despite the costs of this effort (21). If such dynamic, ongoing involvement were observed, it would suggest that amygdala and NAcc activity can work in a coordinated manner with frontoparietal cortical regions (i.e., prefrontal and parietal cortical regions that are frequently coactivated during cognitive tasks) (8, 17, 31–34) to sustain cognitive effort.

This possibility becomes more plausible when considering the extensive anatomical circuits that link these subcortical regions with the prefrontal cortex (PFC) and other cortical regions (35–39). For instance, the basolateral amygdala (BLA) has direct reciprocal connections with the orbital, medial, and ventrolateral PFC, as well as indirect connections to these regions via the mediodorsal thalamus (39–41). The NAcc receives direct input from the ventromedial prefrontal cortex (VMPFC) and anterior insula (aIns) and projects back to the PFC indirectly via loops involving the ventral pallidum, mediodorsal thalamus, and hypothalamus (35). This integrated cortical–subcortical anatomical architecture (35, 36) could, in theory, allow for ongoing interactions between the amygdala, NAcc, and frontoparietal regions throughout task performance, ensuring that cognitive and value-related activity are effectively coordinated to sustain cognitive effort.

In the current research, we test the hypothesis that the amygdala and NAcc are systematically engaged throughout working memory task performance, operating in parallel with frontoparietal regions to sustain executive control. We test three core questions: i) Does amygdala and NAcc value-coding activity essentially disengage once cognitive effort is initiated, or do they sustain their involvement throughout task execution? ii) Does trial-to-trial variation in their value-coding activity during a task predict frontoparietal engagement and behavioral performance [i.e., reaction times (RTs) on correctly performed trials]? And iii) do the amygdala and NAcc functionally interact with frontoparietal regions throughout task performance, consistent with the idea that they work in an integrated manner?

**Fig. 1.**
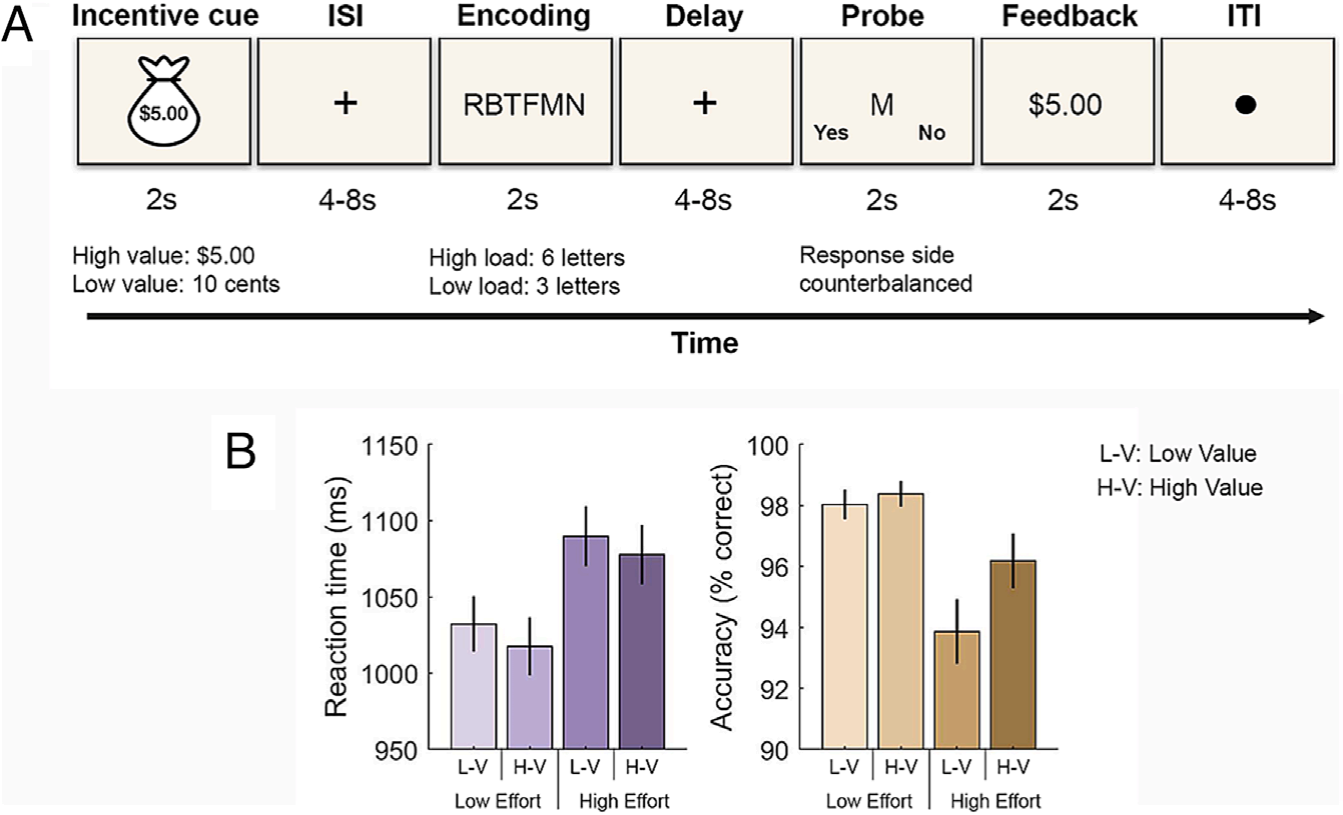
Task design and behavioral performance. (*A*). Trials started with an incentive cue (2 s) that signaled the monetary amount that could be earned contingent on accurate performance. After a variable length fixation period (4 s or 8 s), participants saw a string of three letters (low load) or six letters (high load) to encode into working memory (2 s). Following a variable length delay period (4 s or 8 s), participants reported whether a single letter (probe stimulus) was part of the encoding set (2 s). A feedback screen then indicated if they were correct or not (2 s) and this was followed by an intertrial interval of 4 s or 8 s. (*B*) Behavioral performance (correct reaction time and accuracy) as a function of value and memory load (cognitive effort demands).

To test these questions, we used an event-related functional magnetic resonance imaging (fMRI) design to isolate blood oxygen level-dependent (BOLD) signals (“activity”) at specific time-points within each trial of an adapted Sternberg working memory task (42) (Fig. 1*A*). This task is ideal to address our questions because, unlike other executive control tasks that require only brief cognitive activity (e.g., the Stroop task), it requires a series of distinct cognitive processes that unfold over a more extended time period. Thus, by sustaining cognitive effort we mean maintaining cognitive engagement until each trial is completed, which contrasts with initiating cognitive activity through a decision. Moreover, to disentangle incentive value and cognitive effort demands, they were independently manipulated on a trial-by-trial basis. Finally, by including an incentive cue period and multiple, sequential cognitive processes (memory encoding, memory maintenance, a memory-guided response) that were temporally jittered, we were able to identify whether and when amygdala and NAcc activity was involved while cognitive effort was sustained. To do so, we applied a combination of univariate and multivariate pattern analyses (MVPA) (43, 44), as well as functional coupling analyses (45, 46). Taken together, these methods and analyses allowed us to identify roles for the amygdala and NAcc in supporting effortful cognitive activity.

## Results

We developed and validated an incentivized Sternberg-type (42) working memory task that participants completed while undergoing fMRI acquisition (Fig. 1*A*; see *SI Appendix*, *Results*). On each trial, participants viewed an incentive cue that indicated the potential to earn a +$5.00 (high value) or +$0.10 (low value) incentive for correct responses. After a temporally variable interstimulus interval, participants saw a three-letter string (low working memory load) or six-letter string (high working memory load, held it in mind for the duration of a jittered memory delay period, and then indicated whether a probe letter matched any letter in the previously presented set. At the end of each trial, participants received feedback. Incentive value and working memory load (hereafter “cognitive effort” demands) varied independently across trials in a full factorial design. Participants reported that remembering six-letter strings on high load trials (*M* = 4.67, SD = 1.22) required more effort than remembering three-letter strings on low load trials (*M* = 2.50, SD = 1.00), *t*_35_ = 13.39, *P* < 0.001, Cohen’s *d* = 2.23. All fMRI analyses were preregistered unless otherwise noted as exploratory (*Methods and Materials* and *SI Appendix*). All reported *P*-values are Bonferroni corrected for multiple comparisons [the number of regions of interest (ROIs)], unless otherwise noted as uncorrected.

### Behavioral Results.

Prior studies have consistently shown that motivational incentives can enhance performance on executive function tasks (17, 31, 47–49). Replicating this work (Fig. 1*B*), participants were significantly more accurate on high vs. low incentive value trials (main effect of value: *F*_1, 35_ = 5.96, *P* = 0.02, *η_p_*^2^ = 0.15). In line with prior studies (17, 47, 49), reaction times tended to be faster for high vs. low incentive value trials, although this difference did not reach statistical significance (main effect of value for correct, log-transformed RTs: *F*_1, 35_ = 3.85, *P* = 0.06, *η_p_* ^2^ = 0.10). Performance was also influenced by cognitive effort demands. Participants were more accurate and responded faster on low cognitive effort trials than high cognitive effort trials (main effect of cognitive effort demands on accuracy: *F*_1, 35_ = 14.93, *P* < 0.001, *η_p_*^2^ = 0.30; main effect of cognitive effort demands on RTs: *F*_1, 35_ = 56.58, *P* < 0.001, *η_p_*^2^ = 0.62). There were no significant interactions between incentive value and cognitive effort on accuracy or RTs (*P*’s > 0.05), suggesting that incentive value and cognitive effort demands independently influenced performance.

### Preliminary fMRI Analyses.

As preliminary analyses, we first examined task-related activity in a frontoparietal ROI derived from a meta-analysis of working memory studies (*Methods and Materials*; *SI Appendix*, Fig. S1). Replicating prior work, there was greater frontoparietal activity on high vs. low cognitive effort trials during all task periods. Moreover, in line with prior studies of motivated cognitive control (8, 31), there was greater frontoparietal activity for high vs. low incentive value trials (*SI Appendix*, Fig. S1).

### Univariate Evidence that Amygdala and NAcc Activity Reflects Incentive Value and Cognitive Effort Demands Throughout Task Performance.

**Fig 2.**
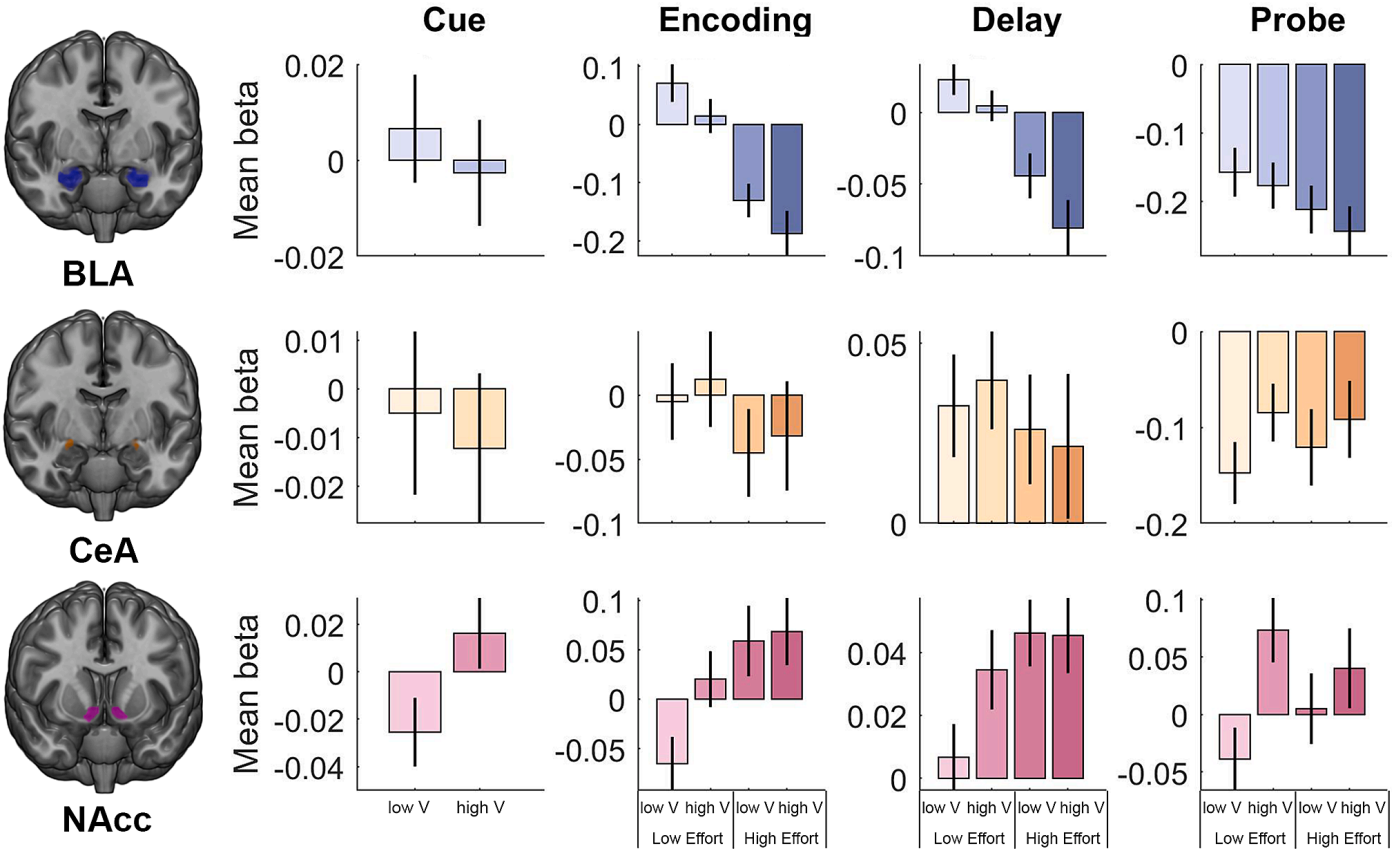
Mean activity in each anatomical ROI during the task. BLA activity demonstrated an effect of incentive value during the encoding and delay periods and an effect of memory load (cognitive effort demands) during all task periods. Central amygdala (CeA) activity was suggestive of general engagement during the delay period, but showed no effect of incentive value or cognitive effort. NAcc activity demonstrated an effect of incentive value during the cue and probe/response periods, and an effect of cognitive effort demands during the encoding and delay periods. Abbreviations: L-V: low value trials. H-V: high value trials.

Do the amygdala (basolateral and central subdivisions) and NAcc essentially disengage when cognitive effort is exerted, or do they play a role in representing the value of sustaining cognitive effort throughout working memory task performance? The results revealed systematic and continuous task involvement, with amygdala and NAcc activity demonstrating distinct, but complementary profiles of engagement (Fig. 2; see also timecourse analysis in *SI Appendix*, Fig. S2).

BLA activity did not reflect incentive value during the cue period (*P* > 0.05), but did exhibit sustained involvement throughout the working memory task. Mean BLA activity reflected the main effect of cognitive effort demands across all task periods (encoding: *F*_1, 35_ = 40.45, *P* < 0.001, *η_p_*^2^ = 0.54; delay: *F*_1, 35_ = 30.53, *P* < 0.001, *η*_p_^2^ = 0.47; probe/response: *F*_1, 35_ = 7.83, *P* = 0.025, *η_p_*^2^ = 0.18) and reflected the main effect of incentive value during the delay period (*F*_1, 35_ = 7.84, *P* = 0.025, *η_p_*^2^ = 0.18). Although BLA activity visually appeared to demonstrate a similar pattern during the encoding period, the main effect of incentive value did not reach the corrected statistical significance threshold (*F*_1, 35_ = 5.49, *P* = 0.075, *η_p_*^2^ = 0.14). Together, these findings indicate that BLA activity reflected the value of cognitive effort especially when working memory was most taxed (the delay period), while also signaling cognitive effort demands throughout all task periods. By contrast, mean CeA activity did not reflect incentive value or cognitive effort during any task period (*P*’s > 0.05).

NAcc activity similarly demonstrated ongoing task involvement, reflecting either incentive value or cognitive effort demands during each task period. Specifically, mean NAcc activity reflected the main effect of cognitive effort demands during the encoding and delay periods (*F*_1, 35_ = 8.96, *p =* 0.015, *η_p_*^2^ = 0.20 and *F*_1, 35_ = 7.48, *P* = 0.029, *η_p_*^2^ = 0.18, respectively) and reflected the main effect of incentive value during the incentive cue (*t*_35_ = 2.63, *P* = 0.037, *d* = 0.46) and probe/response (*F*_1, 35_ = 8.38, *P* = 0.02, *η_p_*^2^ = 0.19) periods. Thus, while BLA activity reflected incentive value while information was maintained in working memory, NAcc activity reflected incentive value when a memory-guided action was generated, suggesting complementary roles in supporting effortful cognitive task performance. Notably, NAcc activity still reflected value during the probe even when controlling for value processing during the immediately following feedback period (*SI Appendix*, Fig. S3*A*). Moreover, NAcc activity was not sensitive to value at the time of feedback (*SI Appendix*, Fig. S3*B*). Together, these findings suggest that probe-related value effects were not driven by feedback period value processing. There were no value x cognitive effort interactions for any ROI (all *P’*s > 0.05).

In summary, the two crucial components of the value of control—incentive value and cognitive effort demands—were dynamically represented in BLA and NAcc activity during specific periods of the working memory task. Interestingly, NAcc activity exhibited the predicted pattern of a greater response for high vs. low incentive value trials, but also demonstrated a greater response for high vs. low cognitive effort demands (Fig. 2). In contrast, BLA activity demonstrated the opposite pattern (Fig. 2).

### Convergent Multivariate Evidence that Amygdala and NAcc Activity Patterns Represent Incentive Value and Cognitive Effort Demands During Task Performance.

Univariate analyses demonstrated a general association between brain signal magnitude and manipulations of incentive value and cognitive effort demands. But do BLA and NAcc multivariate voxel patterns during the task also distinguish whether an individual is in a high vs. low incentive value state, or a high vs. low cognitive effort state? We used MVPA with a leave-one-run-out cross-validation approach to directly test this question about the specific representations supported by amygdala and NAcc activity during each period of task performance.

The MVPA results provided convergent and complementary evidence for BLA and NAcc involvement in sustaining cognitive effort. Consistent with the univariate results, BLA voxel patterns represented incentive value and cognitive effort demands during the encoding and delay periods, whereas NAcc voxel patterns represented incentive value during the probe/response period.

**Fig. 3.**
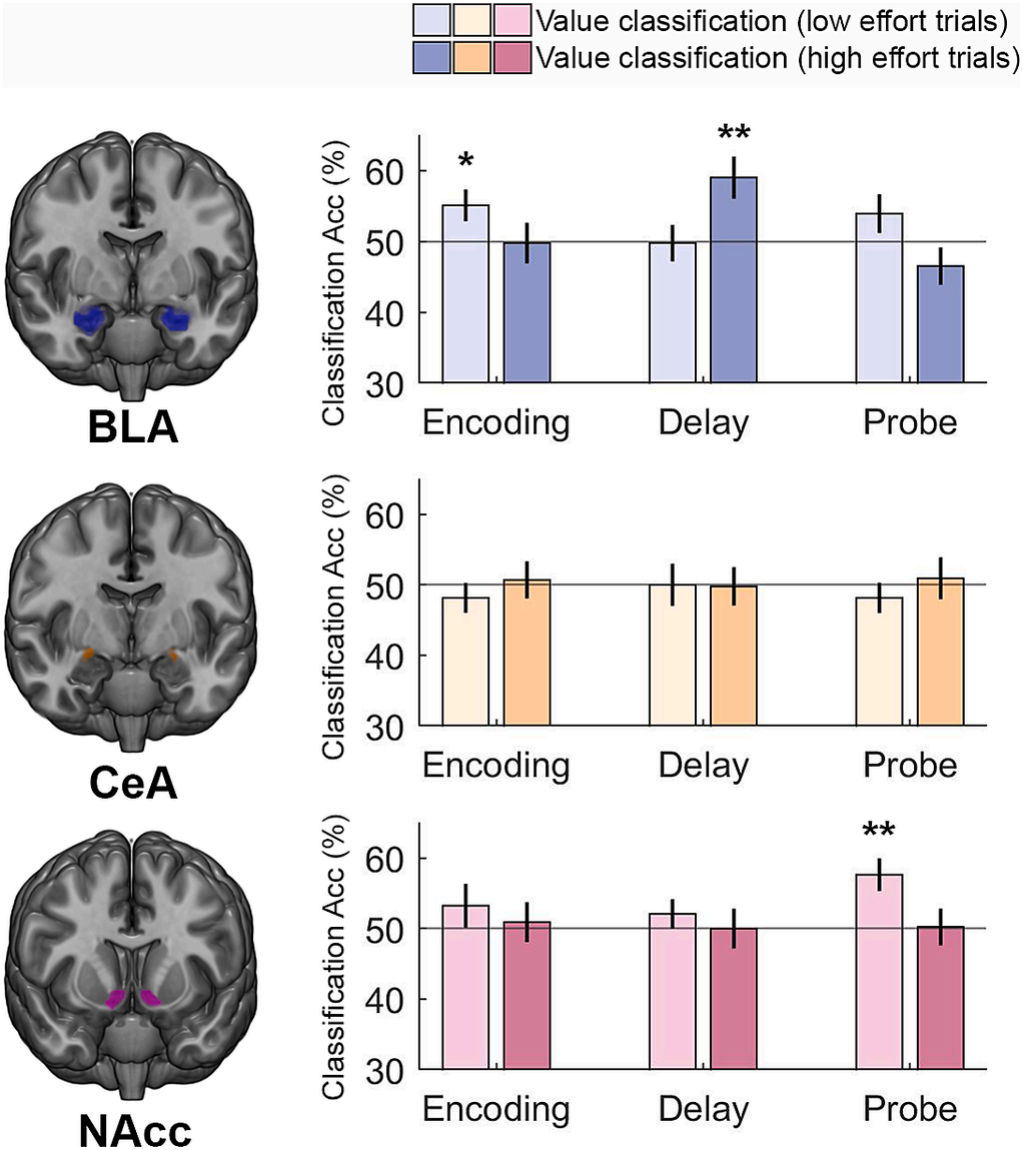
Multivariate classification of high vs. low incentive value during working memory task performance. Incentive value could be classified from BLA voxel patterns during the encoding period of low cognitive effort trials, and from BLA voxel patterns during the delay period of high cognitive effort trials. Incentive value could be classified from NAcc voxel patterns during the probe/response period of low cognitive effort trials. *P < 0.05, Bonferroni corrected, **P < 0.01, Bonferroni corrected.

**Fig. 4.**
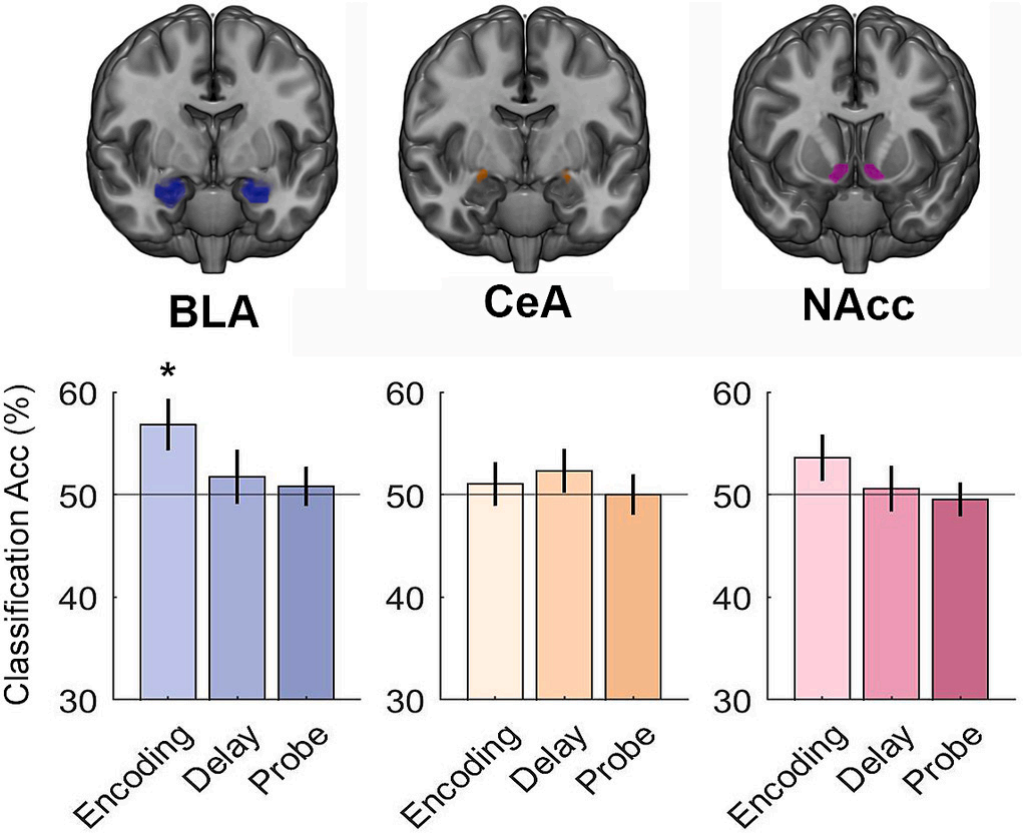
Multivariate classification of high vs. low cognitive effort demands (collapsed across value) during working memory task performance. Significant classification of cognitive effort demands was obtained using BLA voxel patterns from the encoding period. *P < 0.05, Bonferroni corrected.

In these analyses, however, it was important to consider high and low cognitive effort trials separately (Fig. 3*A*) rather than consider the effect of incentive value collapsed across cognitive effort demands (*SI Appendix*, Figs. S4 and S5). Specifically, an exploratory analysis revealed that incentive value could be classified from BLA voxel patterns during the encoding period of low cognitive effort trials (mean = 55.09%, *t*_35_ = 2.25, *P* = 0.046, *d* = 0.38) and during the delay period of high cognitive effort trials (mean = 59.03%, *t*_35_ = 3.03, *P* = 0.007, *d* = 0.51). This suggests that BLA voxel patterns may reflect incentive value during different task periods depending on cognitive demands—earlier during easier trials and later when the task is more difficult. MVPA further revealed that cognitive effort demands were also represented in BLA voxel patterns during the encoding period (Fig. 4*A*; mean: 56.83%, *t*_35_ = 2.71, *P* = 0.016, *d* = 0.45). An exploratory analysis revealed that cognitive effort demands were also represented in BLA voxel patterns and during the delay period of high value trials (mean: 57.41%, *t*_35_ = 2.58, *P* = 0.021, *d* = 0.43). Neither incentive value nor cognitive effort demands could be classified from CeA voxel patterns (all *P*’s > 0.05), so we did not examine this region in subsequent multivariate analyses.

An exploratory analysis revealed that incentive value could be classified from NAcc voxel patterns during the probe/response period of low cognitive effort trials (mean = 57.64%, *t*_35_ = 3.27, *P* = 0.004, *d* = 0.55). Notably, classification of value could be dissociated from classification of the motor response (left vs. right) during the probe/ response period. Whereas incentive value but not the motor response could be classified from NAcc voxel patterns, the motor response but not incentive value could be classified from somatomotor voxel patterns (*SI Appendix*, Fig. S6). Cognitive effort demands, however, could not be classified from NAcc voxel patterns (all *P*’s > 0.05). Together, these findings offer convergent evidence that BLA and NAcc activity patterns are involved in representing the value of sustaining cognitive effort throughout task performance.

### Value Representations Evolve from Cue to Task Periods.

Are the value representations apparent in BLA and NAcc activity patterns during the task a simple carry-over of value representations evoked during the incentive cue period, or are they distinct representations that may reflect cognitive demands? A pattern similarity analysis (50) revealed that value-related voxel patterns during the cue period were largely distinct from value-related patterns during the task (*SI Appendix*, Fig. S8*A*). For the BLA, there were only small, although statistically significant, voxel pattern correlations between the cue and each task event (cue-encoding: mean *r* = 0.08, *P* = 0.048; cue-delay: mean *r* = 0.08, *P* = 0.012; cue-probe/response: mean *r* = 0.11, *P* = 0.014). For the NAcc, the voxel pattern correlations were not statistically significant or were marginal (cue-encoding: mean *r* = 0.09, *P* = 0.11; cue-delay: mean *r* = 0.11, *P* = 0.067; cue-probe/response: mean *r* = 0.11, *P* = 0.052). These low correlations between cue and task value patterns are consistent with the idea that value representations evolve after the introduction of cognitive demands (for convergent evidence, see cross-validated cross-classification analyses in *SI Appendix*, Fig. S9, and within-trial cue-task relationships in *SI Appendix*, *Results*). Once task performance began, value representations became more stable in both regions, with relatively strong value-related voxel pattern correlations from encoding to delay (BLA: mean *r* = 0.48, *P* < 0.001; NAcc: mean *r* = 0.48, *P* < 0.001) and from delay to probe/response (BLA: mean *r* = 0.48, *P* < 0.001; NAcc: mean *r* = 0.45, *P* < 0.001).

Control analyses ruled out alternative explanations based on task timing or trivial shifts in value coding (*SI Appendix*, Fig. S8 *B* and *C*). Because incentive value and cognitive effort regressors shared identical timing, replacing value patterns with effort patterns should produce similar correlations if task timing drove these results. However, this was not observed (*SI Appendix*, Fig. S8*B*). The results also held when restricting the analysis to participants who showed reliable cue and task value decoding, indicating that the results were not driven by a shift from a lack of value coding during the cue to the presence of value coding during the task (*SI Appendix*, Fig. S8*C*).

### Multivariate Value Coding Is Associated With Frontoparietal Engagement and Working Memory Performance.

**Fig 5.**
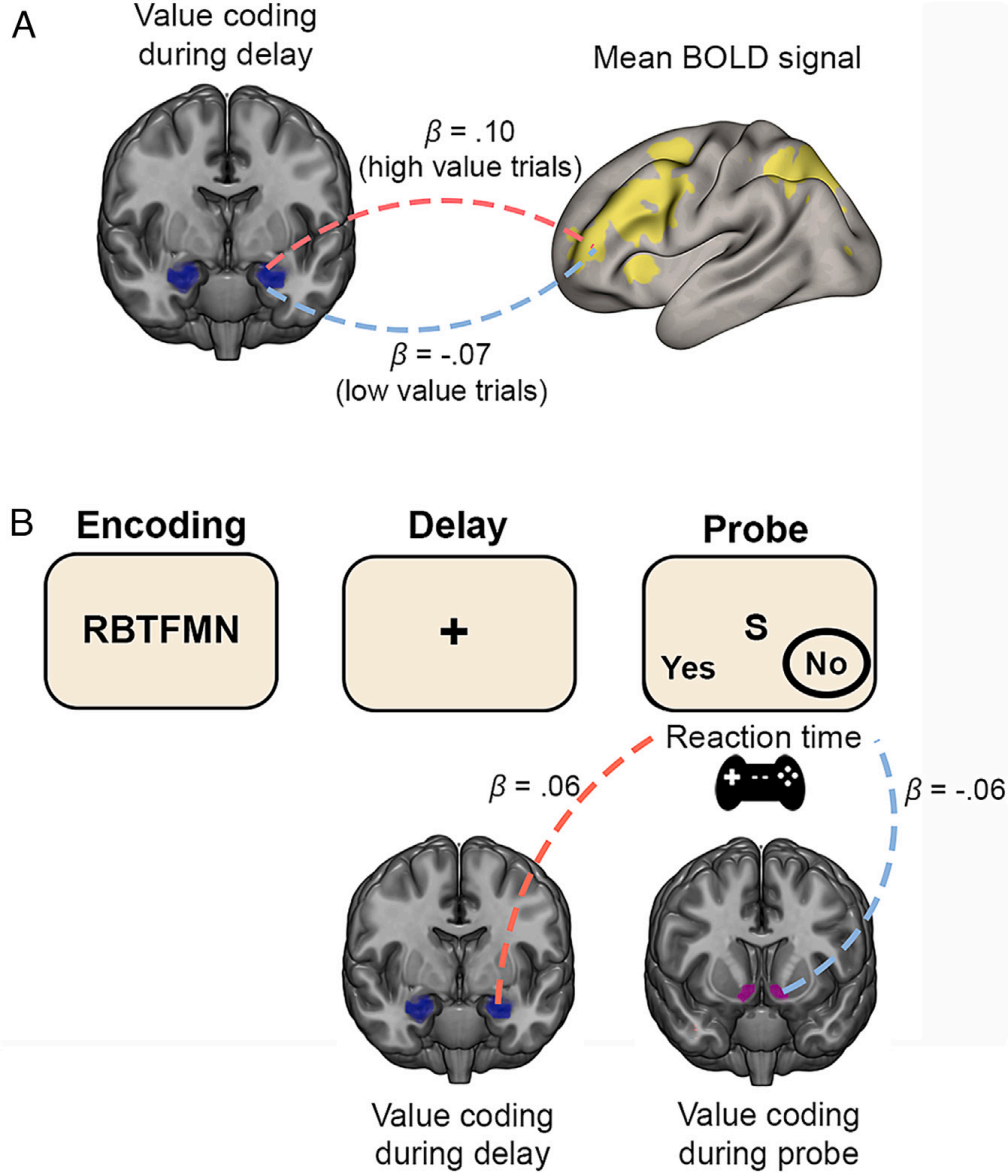
Associations between trial-level BLA and NAcc multivariate value coding and frontoparietal engagement and reaction time (RT). (*A*) Trial-to-trial value coding in the BLA during the delay period was positively associated with mean BOLD signal in the frontoparietal working memory ROI on high value trials and negatively associated with mean BOLD signal in the frontoparietal working memory ROI on low value trials. (*B*) On high value trials, value coding in the BLA during the delay period was associated with slower responses, whereas value coding in the NAcc during the probe period was associated with faster responses.

Do these BLA and NAcc value representations that emerge during the task have relevance to the engagement of frontoparietal working memory regions or to working memory performance (i.e., RTs on correct trials)? In these analyses, we focused on BLA value coding during the memory delay period (when value classification was significant and the strongest) and NAcc value coding during the probe/response period (when value classification was significant). We first quantified the extent to which value coding was expressed on each trial in BLA and NAcc multivariate patterns (*Methods and Materials*). On high incentive value trials, BLA multivariate value coding during the delay period was positively associated with mean BOLD signal in the frontoparietal working memory ROI (Fig. 5*A*; mean *β* = 0.10, *t*_35_ = 3.15, *P* = 0.007, *d* = 0.53). Conversely, on low incentive value trials, BLA value coding showed a negative association with mean frontoparietal BOLD signal, though this did not reach the corrected statistical significance threshold (Fig. 5*A*; mean *β* = −0.07, *t*_35_ = 2.30, *P* = 0.055, *d* = 0.38). There were no significant associations, however, between BLA value coding during the cue period and frontoparietal BOLD signal, or between cue- or task-related NAcc value coding and frontoparietal BOLD signal (all *P*’s > 0.05).

Task-related value coding was also relevant to working memory performance. BLA value coding during the delay period predicted slower correct RTs on high value trials (Fig. 5*B*; mean *β* = 0.06, *t*_35_ = 2.83, *P* = 0.015, *d* = 0.47), potentially suggesting that the BLA promotes more cautious responding to avoid errors (see interpretation in *SI Appendix*, *Results*). On the other hand, NAcc value coding during the probe/response period tended to be associated with faster correct RTs on high value trials, but this did not reach the corrected statistical significance threshold (Fig. 5*B*; mean *β* = −0.06, *t*_35_ = 2.10, *P* = 0.086, *d* = 0.35). There were no other associations between value coding and RT (all *P*’s > 0.05). Interestingly, BLA delay value coding showed a weak, but statistically significant negative correlation with NAcc probe/response value coding on high value trials (mean *r* = −0.08, *P* = 0.005) and both predicted RT when included in the same linear regression model (BLA: mean *β* = 0.06, *P* = 0.011; NAcc: mean *β* = −0.06, *P* = 0.037). This potentially suggests that they exert independent influences on performance.

An exploratory univariate analysis revealed that mean activity in the BLA and NAcc also predicted frontoparietal engagement and RTs (*SI Appendix*, *Results*), providing convergent evidence for the relevance of BLA and NAcc activity in task involvement. Beyond these within-person associations, we also examined between-person associations between brain activity and performance (*SI Appendix*, *Results*).

### The Amygdala and NAcc Functionally Interact with Frontoparietal Cortical Regions.

If the BLA and NAcc work in concert with frontoparietal regions to sustain cognitive effort, we might expect evidence of functional interactions—often operationalized as significant functional coupling in their activity over time (45, 46). We tested this by examining functional coupling between BLA and NAcc BOLD signals and the BOLD signals of voxels located within two preregistered ROIs: i) a Neurosynth-derived meta-analytic ROI encompassing frontoparietal regions associated with working memory; ii) an ROI encompassing the dorsolateral PFC and adjacent territories derived from a meta-analysis of executive components of working memory (51). We first tested for value-related modulations of functional coupling using a generalized psychophysiological interaction (gPPI) analysis (46), which quantified the extent to which BLA and NAcc BOLD signals show stronger coupling with working memory cortical BOLD signals on high vs. low incentive value trials. There were no significant value-related modulations of functional coupling with voxels located within the a priori defined ROIs.

**Fig. 6.**
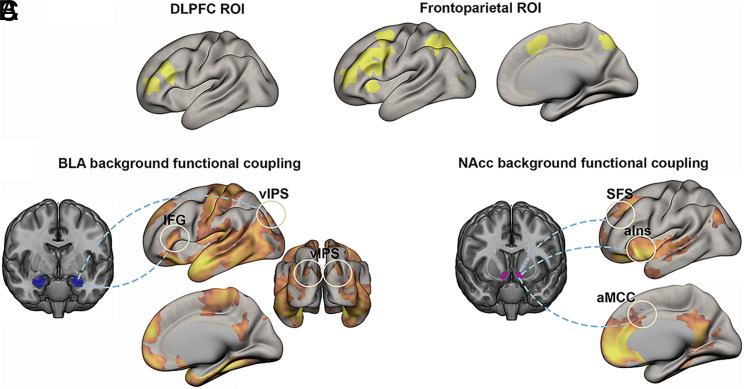
Background functional coupling patterns of BLA and NAcc BOLD signals. (*A*) Meta-analysis derived DLPFC and frontoparietal working memory ROIs that served as search volumes. (*B*) BLA wholebrain functional coupling patterns (*Z* > 3.1, *P* < 0.05 FWE corrected). (*C*) NAcc wholebrain functional coupling patterns (*Z* > 3.1, *P* < 0.05 FWE corrected). Highlighted are the voxels showing significant functional coupling that are located within the dorsolateral or frontoparietal working memory ROIs. Please note that the highlighted SFS on the *Left* side is for illustration purposes, given that the *Right* SFS (where significant voxels were located) is not visible. Abbreviations: aINS, anterior insula; aMCC, anterior mid-cingulate cortex; DLPFC, dorsolateral prefrontal cortex; IFG, inferior frontal gyrus; NAcc, nucleus accumbens; superior frontal sulcus, SFS; vIPS, ventral intraparietal sulcus.

Given that participants reported high motivation to perform well on all trials, it might be the case that amygdala and NAcc activity is significantly coupled with frontoparietal cortical regions across the entire task, irrespective of trial type. Thus, in an exploratory analysis we examined “background” functional coupling (45) across all task periods and trial types—that is, functional coupling that reflects a combination of intrinsic network dynamics and the influence of the overall task context (Fig. 6). This analysis tests the possibility of a tonic (rather than dynamic value-specific) functional relationship between subcortical valuation regions and frontoparietal cortex. While prior work has documented stable patterns of subcortical-cortical coupling during resting states, the key question addressed here is whether the amygdala or NAcc show such functional coupling with cortical regions that have been linked to working memory functions. BLA BOLD signal was significantly coupled with BOLD signal in voxels located within the dorsolateral PFC ROI (*P* < 0.05 family-wise error (FWE) small-volume corrected), with peaks in the bilateral inferior frontal gyrus; x = -53, y =27, z = 17; x = 52, y =24, z = 17), and significantly coupled with voxels located within the frontoparietal ROI (*P* < 0.05 FWE small-volume corrected), with peaks in the bilateral ventral intraparietal sulcus (x = 32, y = −83, z = 37; x = −26, y = −86, z = 29). NAcc BOLD signal was significantly coupled with BOLD signal in voxels located within the frontoparietal ROI (*P* < 0.05 FWE small-volume corrected), with peaks in the bilateral aIns (x = 29, y = 21, z = −3; x = −26, y = 19, z = -3), anterior mid-cingulate cortex (aMCC) (x = −3, y = 21, z = 37), and superior frontal sulcus (x = 17, y = 36, z = 40). These findings provide support for the idea that the BLA and NAcc may functionally interact with frontoparietal regions throughout the task.

Although CeA activity did not show evidence of value coding, it may nevertheless play a key role in information flow to, and from, frontoparietal regions. Consistent with this notion (*SI Appendix*, Fig. S10), CeA BOLD signal was significantly coupled with BOLD signal in voxels located within the DLPFC ROI (*P* < 0.05 FWE small-volume corrected), with peaks in the right inferior frontal sulcus (x = 46, y = 19, z = 26; x = 46, y = 27, z = 20) and left inferior frontal gyrus (x = −46, y = 21, z = 20), and was coupled with voxels located within the frontoparietal working memory ROI (*P* < 0.05 FWE small-volume corrected), with a peak in the left aIns (x = −26, y = 19, z = −3). Thus, CeA activity may contribute to large-scale network interactions involving frontoparietal regions.

### Exploratory Analyses of Activity in Cortical Regions Implicated in Motivated Cognitive Control.

To link these findings with existing work that has identified specific cortical contributions to motivated cognitive control (17, 32, 33, 52), we performed exploratory analyses using ROIs for the VMPFC, the aMCC (sometimes referred to as the dorsal anterior cingulate cortex), and the aIns. Cortical regions showed distinct patterns of activity compared to the BLA and NAcc. Univariate results revealed that VMPFC findings somewhat resembled those of the BLA, while aMCC and aINs findings somewhat resembled those of the NAcc (*SI Appendix*, Fig. S11), however, MVPA revealed that cortical voxel patterns represented cognitive effort demands but not incentive value during each period of the task (*SI Appendix*, Fig. S11). These results, again, support the importance of considering subcortical, in addition to cortical, contributions to sustaining cognitive effort.

## Discussion

Accomplishing important goals often requires sustained cognitive effort. The current results reveal the systematic involvement of the amygdala and NAcc activity throughout an incentivized working memory task. Convergent univariate and multivariate analyses revealed that both the BLA and NAcc represented incentive value, cognitive effort demands, or both, during every period of the task. These regions may thus contribute to ongoing evaluations of whether it is worthwhile to continue exerting cognitive effort from moment to moment (21).

This task-involvement was meaningful as evidenced by trial-to-trial associations between multivariate value coding in these regions and frontoparietal cortical engagement as well as working memory performance. Finally, in line with integrated anatomical connections of these circuits (35–41), amygdala and NAcc activity was correlated with frontoparietal activity, consistent with the idea that they may operate together to sustain cognitive effort. Together, these findings significantly extend prior work (17–20, 47, 53–56) by providing insights into the moment-to-moment contributions of BLA and NAcc representations to sustaining effortful cognitive activity.

Value-based frameworks including the EVC framework (1, 2, 8–14) have recently offered a compelling account of how motivational processes can guide decisions about initiating cognitive effort. The current findings, by demonstrating that the amygdala and NAcc are not only involved before cognitive effort is exerted, but remain continuously engaged throughout a working memory task, suggest that “hot” subcortical motivational processes may be even more of an inherent component of “executive functions” than previously recognized. The current findings may also have implications for expanding the EVC framework to emphasize the dynamic and distributed nature of value signals supporting cognitive effort.

A key contribution of this study is the demonstration of distinct roles of BLA and NAcc activity in supporting incentive value as working memory demands unfolded within each trial. Specifically, BLA activity correlated with value during both the memory encoding and delay periods, whereas NAcc activity correlated with value only during the probe/response period. The BLA, with its reciprocal connections to multiple PFC regions (39, 40), may be relatively more involved in signaling the value of attending to and maintaining task-relevant information in mind (57). By contrast, the NAcc—long characterized as a “limbic-motor interface”—may be relatively more involved in translating anticipated rewards into overt action (27–29, 58, 59). Thus, the willingness to exert cognitive effort in a sustained manner may depend on value signals that are distributed across multiple brain regions and that dynamically emerge according to the cognitive processes that are currently required for effective task performance.

We have interpreted the observed value signals as being centered on specific cognitive control operations. However, it is important to consider an alternative possibility: that these signals reflected an outcome-centered valuation, similar to “state values” in the reinforcement learning framework (60). In the context of our task, a state value signal would reflect a combination of the future reward at stake and the probability of its attainment, which would evolve across a trial as individuals infer their likelihood of responding correctly based on the revealed working memory load and other relevant internal contextual signals. The key point is that a state value signal—even if updating with new information—should be continuously present across the trial. Contrary to this idea, neither BLA nor NAcc activity exhibited this pattern, but instead correlated with temporally selective value signals aligned with specific cognitive operations. Thus, rather than representing state values (and passing this information to other regions for further use), these regions appear to evaluate the worth of engaging particular control processes from moment to moment. That being said, there is evidence to suggest that these regions may also contribute to state value signals in some contexts (25, 61, 62).

The findings reported here are distinct from, but complementary to, influential gating accounts of working memory (63, 64). These accounts and findings (34, 65, 66) address how midbrain dopamine neurons and the dorsal striatum “gate” specific stimuli into, or out of, working memory based on predicted reward value. Thus, gating accounts pertain to which of several stimuli are represented in working memory at any given time, but they do not address why individuals sustain effort in using working memory at all. Our results suggest that BLA and NAcc activity may generate ongoing value and effort-related signals that can provide a continuous motivational context that guides and supports working memory use in general. This motivational context may potentially generate input that leads to the well-documented increase in frontoparietal activity under incentivized conditions, independent of the specific content being maintained (8, 31).

In addition to the strengths of this study, there are some limitations and open questions to address in future research. First, the incentivized working memory task was deliberately optimized to examine how amygdala and NAcc activity contribute to sustaining cognitive effort, rather than to decisions about whether to exert effort at a later time (the focus of prior studies). While this limits direct comparisons of value signals before and during task execution, it was essential for isolating the mechanisms of interest. Second, future work might unravel the functional role of CeA activity, which unlike BLA and NAcc activity, did not respond to the value and cognitive effort manipulations. One possibility is that, during cognitive tasks, CeA activity serves to mobilize appropriate physiological states that can help to maintain heightened attention and readiness to respond (26). Third, future work could examine the extent to which similar neural mechanisms operate during real-world tasks that involve persistence over longer timescales. Based on our findings, it could be that subcortical value signals play a more prominent role in real-world cognitive task performance than traditionally thought. Finally, further work could assess the generalizability of these findings to other forms of motivation (e.g., social incentives, intrinsic motivation, losses), to other executive functions (e.g., response inhibition), and to other participant populations (e.g., older adults or clinical populations).

In summary, these findings support an account in which the amygdala and NAcc play more sophisticated and sustained roles in effortful cognitive activity than previously considered. Thus, cognitively effortful behaviors may rely on coordinated contributions from these subcortical valuation regions as well as frontoparietal regions. Understanding the dynamic interplay between motivational and cognitive processes may provide insights about how people perceive the value of effort and their likelihood of persisting with challenging cognitive tasks.

## Methods and Materials

### Subjects.

Given our preregistered aim to sample 40 individuals, we oversampled to account for data loss due to exclusions and scanned 47 healthy, right-handed, native English-speaking participants from Stanford University who had normal or corrected-to-normal vision. All participants were required to have achieved over 88% accuracy (7/8 trials correct) on the last of three rounds of a practice working memory task, which included no incentive conditions. Based on preregistered criteria, we excluded seven participants due to excessive motion (more than 2 mm within-run motion in any direction). Two participants were excluded based on fMRI task performance accuracy that was below the preregistered criterion (85%), and two were excluded because they reported considerable discomfort/pain throughout scanning and demonstrated excessive framewise displacement (FD). Eight additional participants were scanned with a different set of instructions and were not included in the present analyses. The final sample included 36 individuals (36.11% male, 61.11% female, 2.78% nonbinary/transgender; mean age = 23.00, SD = 4.34; 27.78% white, 27.78% mixed, 16.67% East Asian, 8.33% Hispanic/Latino, 2.78% African American, 2.78% South Asian, 2.78% other). The study was approved by the Stanford University Institutional Review Board. Participants provided written informed consent and were financially compensated for their time.

### Transparency and Openness Statement.

Our methods, hypotheses, and analysis plan were preregistered prior to data collection, which took place from January to December 2024. The preregistration, task, anonymized data, analysis scripts are available on the Open Science Framework (OSF): https://osf.io/2n5wd/?view_only=1bfc7845a0cb410baaa14e40c2dda306.

### Experimental Procedure.

Within a week of scanning, participants were provided instructions about the task and performed a practice session in which they were required to reach a criterion of 88% accuracy over the last block of trials (last 8 trials out of 24 practice trials) on a Sternberg-type working memory task with no incentives. In session 2, participants performed the same Sternberg-type working memory task (with novel stimuli) during fMRI scanning, this time including monetary incentives. The task was incentive compatible, meaning that participants received money earned on a randomly selected subset of trials (six total, one from each functional run, three of which were $5.00 trials and three of which were $0.10 trials), on top of base compensation. Prior to scanning, participants performed eight practice trials, one per trial type (4 conditions × 2 cue types). In the fMRI scanner, participants completed six runs of the task, including a short break in between each run. Outside the scanner, participants completed a questionnaire about their experiences during the task, as well as demographics. Finally, participants were debriefed and compensated.

### Behavioral Task.

Participants performed an adapted Sternberg working memory task (42) in which cognitive effort (high vs. low working memory load) and incentive value (high vs. low) were manipulated, in a 2 × 2 factorial design. The task was programmed using PsychoPy. At the beginning of the trial, participants viewed an incentive cue (2 s), which indicated the possibility to earn $5.00 (high value) or $0.10 (low value) if participants responded correctly. To reduce the likelihood that differences in behavior or neural activity were driven by the visual features of the cues rather than the magnitude of the incentives, we used two distinct cues for each incentive condition (there was only one cue for each condition for subjects 1 and 2). The $5.00 cues consisted of a money bag with “$5.00” written in text or three horizontal lines, and the $0.10 cues consistent of a money bag with “$0.10” written in text or one horizontal line (note that neither the amygdala nor NAcc represented the visual features of the cues, independent of value; *SI Appendix*, Fig. S12).

Presentation of the incentive cue was followed by a jittered fixation period (denoted by a centrally presented cross; 4 or 8 s), supporting examination of brain activation representing value during the task period, uncontaminated by brain responses to the cues themselves. Next, participants viewed a three-letter string (e.g., “J _ _ L _S”) or 6-letter string (e.g., “V T M H K F”) of consonants (2 s; encoding period). The spatial layout was identical across load conditions, with empty positions occupied by an underscore in the low load condition, minimizing confounds related to low-level perceptual details, spatial attention demands, and potential eye movements. The string was followed by a jittered delay period during which participants held the string in mind (4 or 8 s). The jittering of the delay enabled us to separate brain responses during the different task periods, and made each task component less predictable, thereby increasing the need to pay close attention to each task element and to actively rehearse the memory set during the delay period (which lasted for a variable interval). Next, during the probe period, a single letter was presented in the center of the screen (2 s) and participants indicated with a button press whether it matched any of the letters in the encoding set with a “yes” or “no” response. The probe stimulus was always presented in a central spatial location and did not match the spatial location of the target stimulus during the encoding period, in order to ensure that participants could not rely on perceptual processing to make the correct response. To maintain participants’ attention and prevent any type of spatial response bias, the positions of the yes/no response options were laterally counterbalanced (i.e., “yes” appeared on the left of the screen on half of the trials and on the right on the other half). This minimized the possibility that participants could adopt a default bias in response tendency, which might bias classification analyses. Afterward, participants received feedback (2 s) about whether their response was correct (i.e., “$5.00,” “$0.10”) or incorrect (i.e., $0.00). Finally, participants viewed a jittered fixation stop sign, which indicated the end of the trial (4 or 8 s).

The task included 96 trials, divided across six functional runs of 16 trials each (2 value × 2 cognitive effort × 4 repetitions for each run), with 24 trials per trial-type (48 trials per main effect of value and cognitive effort). Trial order was pseudorandomized such that no value or cognitive effort condition appeared more than two times in succession, and all four trial-types were presented an equal number of times within each run, and each was preceded equally often by the other trial-types (except for repeats of the same trial type which occurred less often than a switch to one of the other trial types). Additionally, the letter probe matched the string on half of the trials for each trial-type within each run, and the same letter probe never appeared in succession. Finally, participants received one of two overall trial orders (there were no differences in performance accuracy between them: *P* > 0.05). After scanning, participants were asked to rate the amount of effort it took to remember the three- and six-letter strings on a 7-point scale (e.g., “When you saw three-letter strings, overall, how much effort did it take to remember them?”). This provided an explicit link between the load manipulation and subjective cognitive effort.

### Behavioral Data Analysis.

Behavioral data were analyzed in RStudio (RStudio Team, 2020). Repeated-measures ANOVA tested for mean differences in accuracy and reaction between high vs. low value and cognitive effort conditions using the “afex” package (67).

### fMRI Acquisition.

fMRI data were acquired at the Stanford Center for Cognitive and Neurobiological Imaging using a 3.0T General Electric Discovery MR750 FMRI scanner equipped with a Nova 32-channel head coil. Whole-brain T1-weighted structural scans were acquired using GE’s “BRAVO” sequence and the following parameters: 186 slices; time to repetition (TR) = 6.4 ms, echo time = 2.6 ms, flip angle = 12°, voxel size = 0.9 mm^3^, acquisition matrix = [256, 256]. For each functional run, 46 slices of T2*-weighted echoplanar images were acquired in an interleaved order from inferior to superior with the following parameters: TR = 2 s, echo time = 25 ms, flip angle = 77°, voxel size = 2.9 mm^3^, acquisition matrix = [80, 80]. During each functional run, 220 volumes were acquired. The first 8 volumes were discarded to allow for T1 equilibration effects.

### fMRI Preprocessing.

Functional data analysis was performed using SPM12 (http://www.fil.ion.ucl.ac.uk/spm12). Timeseries data were realigned using a two-pass procedure in which each image was realigned to the first image of each run and then all images were realigned to a mean image across all runs. The anatomical scan was coregistered to the mean functional image. Data were then slice-time corrected (to the first slice). For ROI-based univariate analyses and MVPA, native space, unsmoothed data were used to preserve fine-grained spatial patterns and mitigate noise from adjacent white matter and cerebrospinal fluid (CSF) introduced by normalization and spatial smoothing. For whole-brain searchlight analyses and functional coupling analyses, the data were normalized by first segmenting the anatomical image into gray matter, white matter, CSF, bone, soft tissue, and air/background using a nonlinear deformation field for mapping onto template tissue probability maps (68). Then, the functional images were subsequently normalized to the Montreal Neurological Institute space by applying the forward deformation parameters that were obtained from the segmentation procedure.

### Definition of ROIs.

A priori defined ROIs for the BLA, CeA, and Nacc were obtained from the CIT168 high-resolution, probabilistic atlas of the amygdala and subcortical structures (69, 70). The frontoparietal ROI was derived from a Neurosynth (71) association map of the search term “working memory.” For additional details see *SI Appendix*, *Supplementary Methods*.

To improve data quality and regional specificity, we created subject-specific ROIs by reverse normalizing the atlas ROIs to participants’ native space and intersected them with high probability gray matter maps extracted during segmentation (thresholded at 0.7). These ROIs were then applied to unsmoothed, native space data, thus minimizing the influence of noise from adjacent white matter and CSF.

### Univariate fMRI Analyses.

Task-related BOLD responses were estimated by fitting voxel-wise general linear models (GLMs) to time-series data in SPM12. To isolate signal related to incentive value and cognitive effort for each task event, we used a selective modeling approach involving two GLMs to minimize multicollinearity across task regressors (72). For each GLM, the maximum correlation between task-related regressors was *r* = 0.14 (*SI Appendix*, Fig. S13).

GLM1 was used to examine encoding- and probe-related neural activity. The following regressors were specified at event onset and convolved with a canonical hemodynamic response function (HRF): cue (4 s; 2 regressors reflecting high and low incentive value); encoding (2 s; 4 regressors reflecting the 2 incentive value × 2 cognitive effort factorial design; delay (variable duration; 1 regressor); probe (2 s; 4 regressors reflecting the 2 × 2 factorial design). The delay period modeled the average response and did not specify value and cognitive effort conditions, which reduced collinearity with adjacent events and enabled estimation of encoding- and probe-related condition effects.

GLM2 was used to examine delay-related neural activity. The following regressors were specified at event onset and convolved with a canonical HRF: cue (4 s; 2 regressors reflecting high and low incentive value); encoding (2 s; 1 regressor), delay (variable duration; 4 regressors reflecting the 2 × 2 factorial design); probe (2 s; 1 regressor). Prior work has shown that this selective modeling approach is effective for multicomponent working memory task designs and is unlikely to misattribute signal to adjacent events (72).

We modeled each task event using a canonical HRF, because this approach in combination with jittered trial events affords greater statistical power than other approaches (e.g., FIR model) and readily supports inferences about the roles of the amygdala and NAcc during specific periods of the task, based on the unique variance in BOLD signal accounted for by each task event.

Because probe and feedback periods were only separated in time by 2 s, we did not include separate regressors for the feedback period, as noted in our preregistered analysis plan, and consistent with prior work (17). However, the results are highly similar when an additional set of high and low outcome feedback regressors are included (*SI Appendix*, Fig. S3*A*).

In each GLM, all trials were included because very few miss/error trials occurred (number of misses/errors per run of 16 trials: mean = 0.54, SD = 0.49, max = 2) and excluding or modeling error trials separately could potentially introduce bias in the informational content relevant for multivariate classification. The intertrial fixation periods served as an implicit baseline. The following nuisance variables were also modeled to account for non-neural sources of noise: head motion parameters (three rotation and three translation parameters); FD timecourse (73) to capture bulk motion across successive timepoints; high pass filtered anatomical CompCor nuisance signals (six principal components capturing white matter and cerebrospinal fluid signals extracted from structural masks obtained during segmentation); a constant for each run to account for mean activation differences across runs; and a high-pass filter of 128 s was applied to remove low frequency drifts. The temporal structure of the noise was accounted for with a first-order autoregressive (AR1) model.

### Univariate Statistical Analyses.

For each ROI, mean cue-related beta values were submitted to a paired samples *t* test (high vs. low value) and mean task-related beta values were submitted to a 2 × 2 repeated measures ANOVA (e.g., incentive value × cognitive effort the encoding period). In each case, the threshold for statistical significance was set at α = 0.05 (two-tailed), Bonferroni corrected for three ROIs.

### Multivariate Pattern Classification Analyses.

Multivariate classification analyses were conducted using The Decoding Toolbox (44). We trained and tested classifiers in distinguishing high vs. low value trials during the cue period, and high vs. low incentive value (or cognitive effort) trials during each task period, according to a leave-one-run-out cross-validation approach. The analysis used a linear support vector machine (SVM) classifier with a fixed regularization parameter (C = 1) as implemented in LIBSVM, a library for support vector machines (74). Voxel-wise beta coefficient estimates for high and low value (or effort) for each run served as features for the classification analysis.

For the preregistered analyses of classifying the effect of incentive value (collapsed across cognitive effort) and the effect of cognitive effort (collapsed across incentive value), betas for all four trial types were used, with labels specifying the two relevant classes (e.g., for classification of value, the labels would be 1, 1, −1, −1, for the patterns extracted from the following conditions—high value low effort, high value high effort, low value low effort, low value high effort). For the exploratory classification analyses that considered high and low cognitive effort trials separately (or high and low value trials separately), only the betas for the two relevant conditions were used (e.g., for classification of value during high effort trials, the labels would be 1, −1, for the patterns extracted from the high value high effort condition, and low value high effort condition). These analyses used beta maps for each run from GLM1 for classifications of value/effort during encoding and probe and beta maps from GLM2 for classifications of value/effort during the delay period.

Prior to classification, the data were scaled (z-score “all”) across samples within each feature to have zero mean and unit variance. Patterns for n-1 runs were used for training the classifier and the patterns from the left-out run were used for testing the classifier. This was done iteratively until each run served as the left-out run and then a mean cross-validated accuracy score was computed by averaging across the folds. Chance-level classification accuracy was 50% given that there were equal samples for the two categories of each classification.

We used the Same Analysis Approach (75) to rule out the possibility that irrelevant task features may account for our primary findings (*SI Appendix*, Figs. S14–S16).

### ROI-Based Classification Analyses.

We used an ROI approach in which all voxels within an ROI served as the pattern that was used for training and testing the classifier and the analysis yielded a single accuracy value for that ROI. For group analyses, following standard conventions, a one-sample *t*-test was used to compare classifier accuracies with chance level performance (50%). This approach tests the global null hypothesis that no participants show an effect and does not provide population inference given that below-chance classification accuracies are not meaningful (76). The statistical threshold was set at α = 0.05 (one-tailed, given that only above chance accuracy is meaningful) and Bonferroni corrected for multiple comparisons (three ROIs).

### Relationship Between Value Coding and Frontoparietal Engagement and Performance.

Multivariate value coding was quantified as the correlation between the SVM-derived activation pattern (77) and trial-wise beta coefficients (78). Separate linear regressions tested whether trial-level variation in value coding predicted mean frontoparietal ROI activity and RTs (*SI Appendix*, *Supplementary Methods*).

### Functional Coupling Analyses.

To examine functional coupling, we performed a gPPI analysis (46, 79) and a background functional coupling analysis implemented by the CONN toolbox (release 22.a) (80, 81). Both analyses involved a standard denoising pipeline (82–84) to remove potential confounding effects (*SI Appendix*, *Supplementary Methods*).

## Supplementary Material

Appendix 01 (PDF)

## Data Availability

Anonymized behavioral and fMRI data have been deposited in OSF (https://osf.io/2n5wd/) (85). All other data are included in the manuscript and/or *SI Appendix*.
