# Supplementary material for: Contributions of the basolateral amygdala and nucleus accumbens to sustaining not just initiating cognitive effort
Source: Proc Natl Acad Sci U S A. 2026 May 6;123(19):e2601231123. doi: 10.1073/pnas.2601231123 (PMC13167750; doi:10.1073/pnas.2601231123)
Supplement: Supplementary file 1 — Appendix 01 (PDF) [file pnas.2601231123.sapp.pdf]

PNAS

Supporting Information for

Contributions of the Basolateral Amygdala and Nucleus Accumbens to Sustaining Not Just Initiating Cognitive Effort

Matthew L. Dixon, Elizabeth Blevins, Carol S. Dweck, Kai Görgen, & Brian Knutson

*Correspondence should be addressed to Carol Dweck: dweck@stanford.edu; Matthew L. Dixon: dixonm@stanford.edu; Elizabeth Blevins: eblevins@stanford.edu.

This PDF file includes:

Supporting text
Figures S1 to S16

Supporting Information Text

Supplementary methods

Pre-registered Hypotheses (see full preregistration here: https://osf.io/2n5wd/?view_only=1bfc7845a0cb410baaa14e40c2dda306)

Hypothesis 1: Univariate activation for incentive value and cognitive effort will be consistent with prior work.

a) We predict that the amygdala and NAcc will show significant mean activation for high vs low incentive value during the cue period, but show no effect of cognitive effort during the task period.
b) We predict that the frontoparietal working memory network will show significant mean activation for high vs low cognitive effort during the working memory task period and an effect of incentive value during the cue period.
c) We predict that the NAcc but not the amygdala will probably show an effect of incentive value during the working memory period (low certainty prediction)

Hypothesis 2: MVPA will reveal accurate decoding of high vs low incentive value using amygdala and NAcc activation patterns during both the cue period and working memory task period.

a) We predict that there will be significant (above chance) decoding accuracy for high vs low incentive value during both the cue period and working memory period, using distributed activity patterns of the amygdala and NAcc ROIs.

Hypothesis 3: MVPA will reveal significant decoding of cognitive effort using working memory network activation patterns and using amygdala and NAcc activation patterns.

a) We predict that distributed activity patterns in the working memory network can be used to accurately classify high vs low cognitive effort trials during the working memory task period.
b) We predict that distributed activity patterns in the amygdala / NAcc can be used to accurately classify high vs low cognitive effort trials (reflecting their role in processing effort and task context in relation to value computations).

Hypothesis 4: The representation of incentive value by the amygdala and NAcc will show stability from the cue to the working memory task period.

a) Time-resolved decoding analysis. Using a finite impulse response (FIR) model, we will examine decoding accuracy at each 2 second bin of the trial and we predict that above chance classification will be present from the cue period onward.
b) Cross-classification analysis: By training the classifier only on cue-related incentive value patterns, we predict that it will be possible to classify incentive value during the working memory task period.
c) Pattern similarity analysis: We predict that the distributed activation pattern reflecting the high vs low incentive value contrast during the cue period will be significantly correlated with the distributed activation pattern reflecting high vs low incentive value contrast during the working memory period.
d) Single-trial analysis. We predict that the extent to which the classification pattern for high vs low incentive value is expressed during the cue period of each trial will be correlated with the extent to which it is expressed during the task period of each trial.

Hypothesis 5: Trial-by-trial incentive value strength in the amygdala and NAcc will be associated with working memory network activation and behavioral performance (RT).

a) We predict that the extent to which the classification pattern for high vs low incentive value is expressed on each trial will predict trial-by-trial fluctuations in mean activation magnitude in the working memory network on correct trials.
b) We predict that the extent to which the classification pattern for high vs low incentive value is expressed on each trial will probably predict trial-by-trial fluctuations in RT on correct trials (low certainty prediction).

Hypothesis 6: Functional coupling strength between the amygdala / NAcc and the working memory network will increase during high vs low incentive value trials.

a) We predict that a generalized psychophysiological interaction (gPPI) analysis using the amygdala and NAcc as seed regions will show a significant interaction between task context (high vs low incentive value) and functional coupling strength with the working memory network (in particular the dorsolateral prefrontal cortex).

Hypothesis 7: Neural indices of incentive value will predict between-person differences in behavior

a) We predict that between-person differences in classification accuracy of incentive value using amygdala / NAcc activation patterns will be associated with performance (mean RT and mean accuracy).
b) We predict that between-person differences in pattern similarity between incentive value during the cue and task in the amygdala / NAcc will task predict performance.
c) We predict that between-person differences in mean signal in the amygdala will not be associated with performance (but NAcc might show this pattern).

Updates to pre-registered analysis plan

Deviations from preregistration analysis plan
For transparency, we note several minor changes from our pre-registered data analysis plan.

- We planned on including the results from a combined amygdala/NAcc ROI (in addition to the individual ROI results), however, the results were generally similar to BLA (which contributed the most voxels), and therefore did not add anything meaningful, so was not reported.
- We planned on including results from the overall task period (in addition to results for each task period separately), however, the overall task results were not informative given the stark differences observed across the encoding, delay, and probe periods, and therefore did not add anything meaningful and were not reported.
- In addition to motion and framewise displacement regressors to account for noise, the 1st level models also included 6 anatomical CompCor components to further minimize motion-related and non-neural sources of noise. While this step was preregistered for the functional coupling analyses only, we applied it across all analyses to enhance data quality.
- For the pattern similarity analysis, we initially expected to run 9 tests (3 ROIs x 3 tests). However, given the non-significant value coding results for the CeA, it did not make sense to include it in this analysis. We also decided to included more tests to determine the degree of representational stability during the task periods. Thus, we ran and corrected for 10 tests (2 ROI x 5 tests).
- For the trial-level analyses, we planned on defining the multivariate value coding index based on the SVM-derived activation pattern from a classification involving the overall

task period. However, as noted above, we focused on the preregistered analyses of each task period separately and thus used SVM-derived activation patterns from classifications that were significant (i.e., during the delay period for the BLA and during the probe period for the NAcc).

- For the trial-level analysis of relationship to RT, we planned on including an additional follow-up analysis that would have divided participants into two groups (based on the relationship between frontoparietal signal and RT) and then involved conducting separate analyses on these groups for testing the relationship between BLA/NAcc value coding and RT. However, given that the more straightforward analysis including all participants yielded interpretable results and given the complexity of this extra analysis (e.g., uneven subject numbers between groups) it was dropped.
- We planned on visualizing results from a time-resolved decoding analysis (i.e., computing classification accuracies for each 2-sec time bin of each trial). However, given the temporal jitter in our task design, the trial events did not occur at the same time during each trial which made it difficult to link classification accuracy to different task periods, so we decided to drop this visualization.

ROI definition and thresholding

Definition of ROIs

Our primary analyses were focused on a set of *a priori* defined ROIs. The basolateral complex (BLA) and central (CeA) amygdala ROIs were obtained from the CIT168 high-resolution, probabilistic atlas of the amygdala (http://evendim.caltech.edu/amygdala-atlas) (69). This parcellation is based on high-resolution MRI data from 168 neurotypical adult brains and offers probabilistic delineations for amygdala subdivisions. The BLA was composed of the lateral, basolateral, and basomedial nuclei, thresholded at 20% and resampled to 2.9 mm to match the functional data (mean = 80.53 voxels, SD = 9.66). The CeA included the central nucleus and was thresholded at 1% to retain more voxels given its smaller volume, and resampled to 2.9 mm (mean = 12.06 voxels, SD = 2.73). The NAcc ROI was obtained from the CIT168 high-resolution probabilistic atlas of subcortical nuclei (70), thresholded at 25% and resampled to 2.9 mm (mean = 32.64 voxels, SD = 5.11). ROI thresholding choices were determined *a priori* with the goal of balancing anatomical specificity and voxel coverage for reliable signal estimation. The ROIs were derived from a probabilistic atlas, in which lower probability thresholds include voxels with greater anatomical uncertainty and increased overlap between adjacent nuclei. Because the BLA occupies a substantially larger volume than the CeA, a more conservative probability threshold (20%) was applied to the BLA to prioritize anatomical specificity (minimizing overlap with adjacent nuclei) while still retaining adequate voxel coverage. In contrast, given that the CeA is markedly smaller, a more liberal threshold (1%) was used to prioritize sufficient voxel inclusion for better signal estimation, while still preserving relative anatomical specificity. While some studies are interested in the ventral striatum more broadly, our interest was specifically the nucleus accumbens (NAcc). A somewhat more conservative threshold (25%) was therefore selected to target this region and minimize the inclusion of voxels associated with neighboring regions, while still retaining sufficient voxel coverage.

The frontoparietal ROI was derived from a Neurosynth (71) association map of the search term “working memory”, which included 1091 studies. Association maps reflect voxels that are more likely to show activation consistently in studies that mention the search term compared to studies that do not use the term (significant voxels were defined based on a threshold of $q < .01$, false-discovery rate corrected). This map provides greater certainty that these voxels are involved in the cognitive process in question than more traditional analyses that only test whether voxels are activated by a particular cognitive task. The working memory association map was resampled to 2.9 mm (mean = 1602.19 voxels, SD = 171.68) and included canonical working memory regions including the PFC (dorsolateral PFC, inferior frontal sulcus, frontal eye fields), posterior parietal

cortex (intraparietal sulcus, inferior parietal lobule), pre-SMA, dorsal anterior insula, and cerebellum.

Relationship between value representations during the cue and task
To examine the stability of value representations, we extracted the voxel beta coefficients representing the contrast of high versus low incentive value for the cue period and for each subsequent stage of the task. These contrasts reflected the average difference between high versus low incentive value conditions across all trials and are therefore independent of trial- and run-specific noise that can bias similarity estimates. Each contrast map was vectorized and the similarity of cue and task patterns was quantified using Pearson correlation. To assess statistical significance, the correlation values for each participant were Fisher z-transformed (using Fisher's r-to-z transformation for correlation coefficients) to normalize the distribution and then submitted to a one sample *t*-test against 0. This was done separately for each ROI (BLA and NAcc). The threshold for statistical significance was $\alpha = .05$ (two-tailed), Bonferroni corrected for multiple comparisons (2 ROIs x 5 similarity tests).

Relationship between value coding and frontoparietal engagement and performance
We first defined a multivariate value "signature" within each ROI using transformed classification weights (i.e., the SVM-derived activation pattern) derived from the high versus low value classification. The activation pattern quantifies the contribution of each voxel to discriminating high versus low value conditions. For the BLA, we used the activation pattern from the delay period of high-effort trials, corresponding to peak decoding performance. For the NAcc, we used the activation pattern from the probe period of low-effort trials, corresponding to peak decoding performance.

Single-trial voxel-wise beta coefficients were obtained using the least-squares-all (LSA) approach, in which separate regressors modeled the cue and task period (delay or probe period) of each trial, along with the same nuisance regressors as in the primary GLMs. For each trial, we quantified the expression of the value signature by computing the Pearson correlation between the vectorized value signature (within a given ROI) and the vectorized trial-specific voxel pattern during the task period. Prior to computing correlations, the mean ROI signal was regressed out of each voxel to ensure that this measure reflected spatial pattern similarity rather than univariate amplitude differences. This produced a trial-wise index of multivariate value coding strength. We then tested whether trial-level variation in value coding predicted: (i) mean frontoparietal ROI activity; and (ii) RTs. Separate linear regressions were conducted for high and low value trials and for high and low effort trials, including only correct trials. Analyses were performed for each ROI and participant. Resulting regression coefficients were submitted to a group-level one sample *t*-test against 0 (two-tailed, $\alpha = .05$, Bonferroni corrected for 2 ROIs).

Functional coupling analyses
To examine functional coupling, we performed a generalized psychophysiological interaction analysis (gPPI) and a "background" functional coupling analysis implemented by the CONN toolbox (release 22.a). Both analyses involved a standard denoising pipeline to remove potential confounding effects (For additional details see ***SI Appendix*** **methods)**. The following variables were regressed from the data: 6 total anatomical CompCor noise components from white matter and CSF timeseries, SPM covariates that modeled the initial ramping at the beginning of runs (9 components), 6 motion parameters and their first-order derivatives, and linear trends (2 factors) within each functional run. For the background functional coupling analysis, task-related effects were also regressed out, which allows for estimates of functional coupling that persist independently of task-evoked activity (i.e., functional coupling does not merely reflect task-related co-activation). As a simultaneous step, the BOLD timeseries data were high-pass filtered at 0.009 Hz. CompCor noise components within white matter and cerebrospinal fluid were estimated by computing the average BOLD signal as well as the largest principal components orthogonal to the BOLD average and motion parameters. For both analyses, seed region timeseries were extracted from unsmoothed data, and correlated with whole-brain voxel-wise timeseries extracted from spatially smoothed data (4 mm FWHM kernel).

Separately for each seed region (BLA, CeA, NAcc), a gPPI model was defined using seed BOLD timeseries as the physiological factor, boxcar signals characterizing each task condition (high and low value trials) convolved with an SPM canonical hemodynamic response function as the psychological factors, and the product of the two as the psychophysiological interaction terms. Task-dependent functional coupling changes were characterized by the regression coefficient of the psychophysiological interaction terms in each model. We separately examined functional coupling changes during the cue period and during the entire working memory task performance period. Condition regressors were obtained from a simplified GLM in which only the main effect of incentive value was modeled and included the following regressors: cue (2 regressors: high and low value); entire task performance period (2 regressors: high and low value); feedback period (1 regressor).

For the background functional coupling analysis, we computed the Fisher-transformed bivariate correlation coefficients from a weighted general linear model, defined separately for each pair of seed and target areas, modeling the association between their BOLD signal timeseries. Individual scans were weighted by a boxcar signal characterizing each task event/condition convolved with an SPM canonical hemodynamic response function and rectified. Background functional coupling can be thought of as task-modulated spontaneous activity (i.e., intrinsic dynamics that are influenced by the context of performing a task), which includes cognitive processes that are invoked for effective task engagement.

Supplementary results

Within-trial correspondence between cue and task value representations

As reported in the main text, voxel patterns reflecting value demonstrated little stability from the cue through the task period. This was evident in the pattern similarity analysis reported in the main text, as well as in the cross-validated cross-classification results reported in **Fig. S9**. In a complementary analysis, we examined the relationship between value coding strength during the cue and task period of each individual trial. To do so, we first estimated single-trial beta weights using the LSA approach in which each cue and task period were modeled with separate regressors for each trial. We then quantified the strength of value coding during the cue based on the similarity of each cue beta patten to the SVM-derived activation pattern for the cue value classification, thus providing a timeseries of correlation values. Similarly, we quantified the strength of value coding during the task period based on the similarity of each task beta patten to the SVM-derived activation pattern for the task value classification, thus providing a timeseries of correlation values. We used the activation pattern from the delay period of high cognitive effort trials for the BLA (when it showed the strongest, significant decoding accuracy), and the activation pattern from the probe period of low cognitive effort trials for the NAcc (when it showed significant decoding accuracy). Mean ROI signal during the cue and task periods was regressed out prior to computing their similarities with the activation patterns used to define value coding strength. We then examined the relationship between value coding strength during the cue and task periods of correct trials using linear regression, whereby the timeseries of value coding strength during the cue served as the predictor variable and the timeseries of value coding strength during the task served as the outcome variable. This analysis was conducted separately for each participant and for the BLA and NAcc. To assess statistical significance at the group level, the resulting beta values were submitted to one-sample t-tests against 0, with the threshold for statistical significance set at α = .05 (two-tailed), Bonferroni corrected for multiple comparisons (2 ROIs).

The results of this analysis revealed a modest relationship between *trial-to-trial* fluctuations in value coding strength during the cue and task periods. Simply put, when value coding was stronger during the cue period of a given trial, it was also stronger during the task period of the

same trial (BLA: mean $\beta = .21$, $p < .001$, Bonferroni corrected; NAcc: mean $\beta = .13$ $p < .001$, Bonferroni corrected). Notably, these associations are unlikely to be driven by potential confounds (e.g., non-specific effects of trial-level arousal, or noise) for multiple reasons. First, mean BOLD signal within each ROI was regressed out from the voxel pattern during the cue and task periods. Second, if trial-level confounds drive these findings, we should also see a significant relationship after replacing task-related value coding with task-related cognitive effort coding, given that they both share the same timing. However, there was no relationship between the strength of value coding during the cue period and the strength of cognitive effort coding during the task period for either ROI (*p's* > .05, Bonferroni corrected). Thus, these findings are consistent with a meaningful correspondence between cue and task value coding strength *within* each trial. However, the modest size of these correlations is also consistent with the idea that, while value coding is somewhat stable across the task, it also evolves in line with task demands.

Univariate analysis of BLA and NAcc mean BOLD signal associations with frontoparietal engagement and performance
An exploratory univariate analysis examined the relationship between mean BOLD signal in the BLA and NAcc and frontoparietal engagement and RT on each trial. Mean BLA BOLD signal during the delay was negatively associated with frontoparietal signal during the delay period, for all trial types (all mean *β's* < -.17, *p's* < .001, Bonferroni corrected), but was unrelated to RT (all *p*'s > .05, Bonferroni corrected). Thus, mean BLA BOLD signal associations were distinct from the multivariate value coding associations reported in the main text. Conversely, mean NAcc signal during the probe was unrelated to frontoparietal signal during the probe period of any condition (all *p*'s > .05, Bonferroni corrected), but was associated with faster RTs during low cognitive effort trials: mean $\beta = -.064$, $p = 0.002$, Bonferroni corrected). Thus, both mean BOLD signal and multivariate value coding in the NAcc were associated with task performance (in the same direction). Together, these findings reinforce the idea that BLA and NAcc involvement throughout the task is relevant to frontoparietal engagement and behavioral performance.

Interpretation of the Relationship Between BLA Value Coding and Reaction Time
It may seem counterintuitive that BLA value coding activity predicted slower reaction times (RTs) on high value trials, given that higher value is often associated with faster responses. While we remain cautious in interpreting the nature of this relationship, insights from the decision making literature on speed-accuracy tradeoffs may help to contextualize this finding.

In our task, reward was contingent on accuracy but not on response speed (provided that participants responded within the 2s response window), thus incentivizing participants to prioritize accurate performance over response speed. Within drift–diffusion models of decision making (1, 2), responses are generated by accumulating noisy "evidence" until a decision threshold is reached, at which point a response is executed. In a Sternberg working memory task such as ours, probe responses can be conceptualized as an evidence accumulation process in which the stimulus set held in mind is compared with the probe stimulus over time until sufficient evidence is reached to commit to either a "match" or "non-match" decision. On some trials, individuals may be less confident about the information held in mind and may require more time for evidence to accumulate before committing to a decision. We speculate that on some high value trials, BLA value activity may reflect not only the incentive value, but also the uncertainty around making a correct response, and may contribute to a proactive adjustment of decision threshold in a conservative direction. In other words, BLA engagement on high value trials may contribute to a motivational state that promotes more cautious and deliberate responding to avoid costly errors. While this interpretation is speculative, it could be investigated further in future research.

Effect Sizes and Power Considerations
To contextualize and interpret findings, it can be helpful to consider effect sizes and power in addition to whether results were significant or non-significant. Significant univariate effect sizes ranged from $\eta_p^2 = .18$ to .54 (considered to be moderate to large effects). Significant multivariate effects were generally small to moderate and ranged from Cohen's d = .38 to .55. Significant trial-

level effects (e.g., brain-behavior associations) were also small to moderate and ranged from Cohen's d = .38 to .53.

With N = 36, the study was likely underpowered to detect smaller effects, raising the possibility that additional subtle effects went undetected. Sensitivity analyses indicated that, with N = 36, for a 2 x 2 repeated-measures ANOVA (with assumed correlation of .5 between measures, which is reasonable based on the empirical correlation structure observed in our data), we had ~80% power to detect effects of approximately $\eta_p^2 \sim .09$ at $\alpha = .05$ (and $\eta_p^2 \sim .11$ under Bonferroni correction for multiple comparisons). For multivariate analyses, we had ~80% power to detect effects of approximately d ~ .42 for one-tailed, one-sample t-tests at $\alpha = .05$ (and d ~ .51 under Bonferroni correction). For trial-level analyses, we had ~80% power to detect effects of approximately d ~ .48 for two-tailed, one-sample t-tests at $\alpha = .05$ (and d ~ .60 under Bonferroni correction). Thus, it is important to consider the possibility that additional subtle effects went undetected. Importantly, these considerations do not call into question the robustness of the effects that were detected.

How Performance was Related to Task Timing
Accuracy did not vary as a function of ISI (main effect of ISI length: $F_{1, 35} = 2.18$, $p = .15$; mean Acc ISI: 96.1%, mean 8s ISI: 97.1%) or delay length (main effect of delay length: $F_{1, 35} = .23$, $p = .63$; mean 4s ISI: 96.8%, mean 8s ISI: 96.5%), nor was there an interaction ($F_{1, 35} = .77$, $p = .39$). Log reaction times (RTs) on correct trials did not show a main effect of ISI length or delay length (*p's* > .06), but there was a significant interaction effect ($F_{1, 35} = 23.47$, $p < .001$, $\eta_p^2 = .40$), with the slowest RTs occurring on trials in which the ISI and delay were both 4s in duration (i.e., when both intervals were the shortest). Importantly, these timing intervals did not interact with incentive value and cognitive effort, our key variables of interest. There were no significant two-, three-, or four-way interactions (*p's* > .19). Thus, although the temporal structure of the task influenced RTs in general, it was unrelated to our key manipulations and did not affect performance accuracy. These findings suggest that longer intervals within a trial did not promote attentional lapses or performance failures, and that timing factors did not drive performance differences across conditions.

We further examined whether RTs differed between correct and error trials. A repeated-measures ANOVA on mean log RTs did not reveal a difference ($F_{1, 14} = 3.08$, $p = .10$, $\eta_p^2 = .18$). However, given that 21 participants were excluded due to a lack of error trials, we also conducted a linear mixed-effects model with response type (correct vs. error) as a fixed effect and random intercepts for participants. This analysis may be more sensitive given that all trials are modeled for all participants. RTs were slower on error trials than on correct trials ($\beta = -0.061$, $SE = 0.01$, $t = -7.56$, $p < .001$). Given the very low error rate, these slower RTs could have reflected either a rare lapse in attention, or increased uncertainty about the correct response on these trials."

Between-Person Associations Between Brain Activity and Performance
We examined potential associations between performance (mean RT and mean accuracy) and three indices of value-related brain activity: (i) mean ROI signal for the high versus low value contrast; (ii) decoding accuracy for the high versus low value classification; and (iii) the stability of value coding from the cue to task period (indexed with pattern similarity values).

The only significant associations (Bonferroni corrected for 3 ROIs) were negative relationships between BLA decoding accuracy of value during the task (delay period) and mean RT on high value trials ($\beta = -.46$, $t_{35} = -3.02$, $p = .014$, corrected) and on low cognitive effort trials ($\beta = -.46$, $t_{35} = -3.01$, $p = .015$, corrected). All other associations were not significant (all *p*'s > .05). Thus, individuals whose BLA patterns more reliably distinguished high versus low value states overall

were on average faster to respond on these trial types. However, these between-person results should be seen as preliminary until replicated in a larger sample.

Development and Validation of the Incentivized Working Memory Task

The fMRI task design was guided by the data from two online pilot studies that tested for optimal cue features and reliable behavioral differences as a function of value and cognitive effort conditions. For both studies, we recruited participants from Prolific who were between the ages of 18 and 30, living in the United States, spoke English as their native language, and had an approval rate of 98% or higher. In *Pilot Study 1* (N = 45, 55.56% male, 43.44% female; mean age = 24.20, SD = 3.66), we compared two incentive cue formats (abstract circles vs. money bags) and two incentives values ($5.00 vs $0.50). There were no significant main effects of cue visual form on subjective ratings of valence, arousal (i.e., activation/energization), liking, or motivation evoked by the incentive cues (p's > .24; nor were there any interactions between incentive value and cue visual form: p's > .12). This suggests that the two sets of cues could be used to dissociate visual cue features from incentive value, which was crucial for the fMRI experiment. Behaviorally, participants were more accurate and faster on low cognitive effort than high cognitive effort trials (p's < .05). Although participants rated $5.00 cues as more positive, arousing, likable, and motivating than $0.50 cues (all p's < .001), there were no performance differences between high and low value incentive trials (p's > .09).

In *Pilot Study 2* (N = 21, 47.62% male, 47.62% female, 4.76% non-binary; mean age = 25.29, SD = 3.29), we reduced the low incentive to $0.10 and shortened the response window from 4 s to 2 s to reduce fatigue. Participants rated high value versus low value cues as more positive, arousing, likable, and motivating (all p's < .001) and now performed more accurately on high value than low value trials ($p = .04$), with continued effects of cognitive effort, especially on accuracy ($p < .05$). Together, these results validated a task design with appropriate incentive cues (that dissociated visual features from value) and could evoke reliable behavioral effects of value and cognitive effort. The *fMRI version* incorporated both cue types (circle and money bag), $5.00 (high) and $0.10 (low) incentive trials, and a 2-s response window while enabling event-related analyses of amygdala and NAcc activity throughout task execution.

Supplementary References

1. K. Görgen, M. N. Hebart, C. Allefeld, J.-D. Haynes, The same analysis approach: Practical protection against the pitfalls of novel neuroimaging analysis methods. *Neuroimage* **180**, 19–30 (2018).

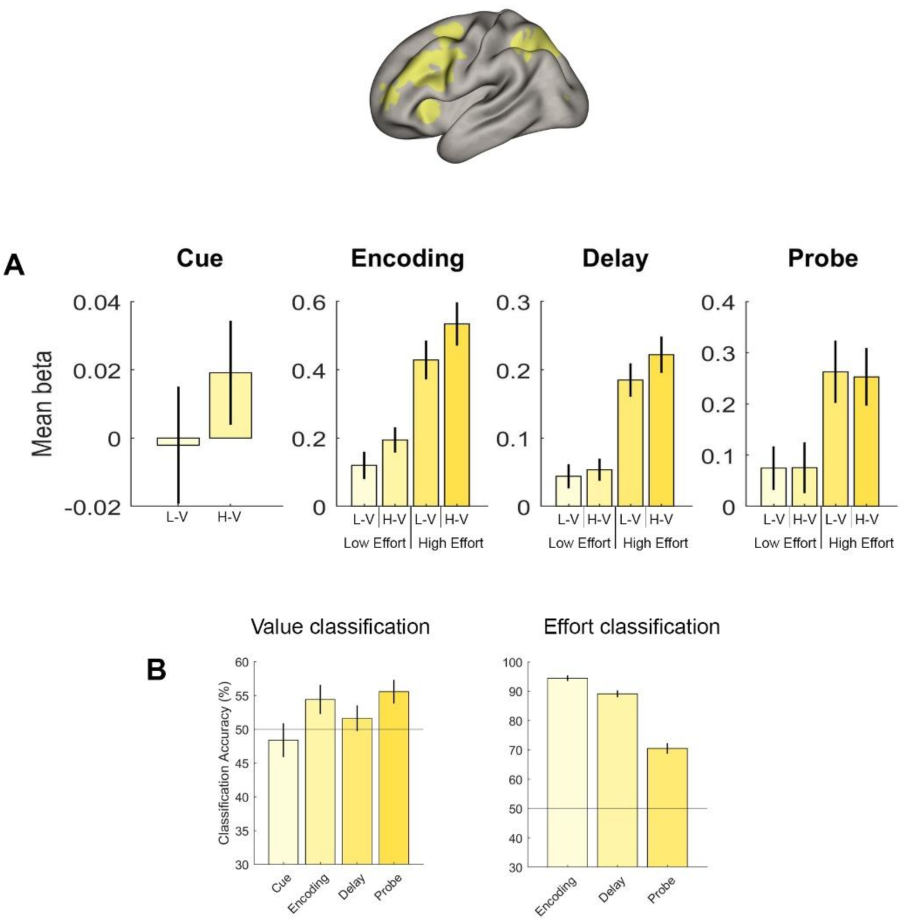

Fig. S1. **Univariate and MVPA results for the Neurosynth-derived frontoparietal working memory ROI**. **(A)** Univariate results. There was a main effect of cognitive effort for all task events (encoding: $F_{1, 35} = 44.72$, $p < .001$; delay: $F_{1, 35} = 54.51$, $p < .001$; probe: $F_{1, 35} = 39.55$, $p < .001$). Frontoparietal BOLD signal also reflected value during the encoding period ($F_{1, 35} = 6.81$, $p = .013$), but not during any other point of the trial, and there were no value x cognitive effort interactions (all *p*'s > .05). **(B)** MVPA results. Cognitive effort could be classified from frontoparietal voxel patterns during each task period with high accuracy (encoding: mean = 94.44%, $t_{35} = 46.60$, $p < .001$; delay (mean = 89.12 %, $t_{35} = 33.23$, $p < .001$; probe: mean = 70.49%, $t_{35} = 11.54$, $p < .001$). Value could be decoded from frontoparietal voxel patterns during encoding (mean = 54.40%, $t_{35} = 2.051$, $p = .024$) and probe (mean = 55.56%, $t_{35} = 3.16$, $p = .002$), but not during the cue (mean = 48.38%, $t_{35} < 1$), or delay period (mean = 51.62%, $t_{35} < 1$). Abbreviations: L-V: low value trials. H-V: high value trials.

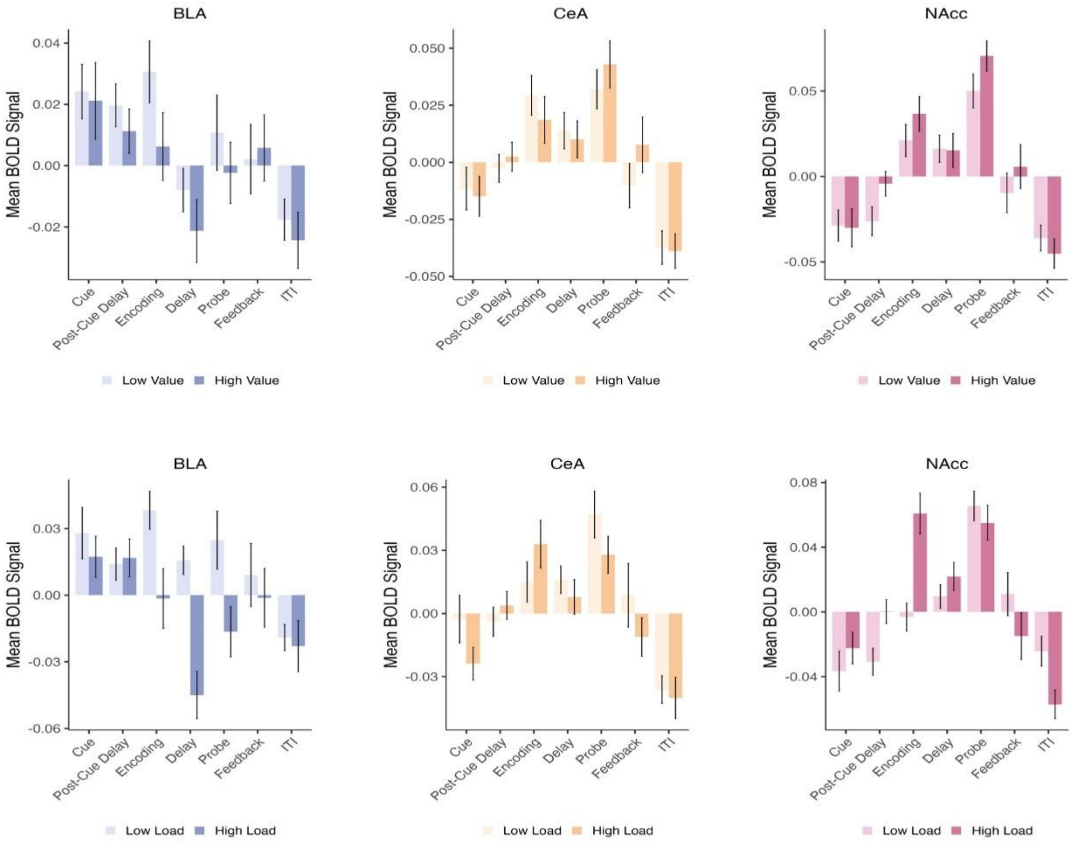

Fig. S2. Raw timecourse-based analysis of mean BOLD signal extracted from ROIs. We conducted an exploratory analysis of the raw timecourses for each ROI to complement the GLM analysis reported in the main text. To visualize the time series data in each ROI across the task as a function of value (top row) and cognitive effort (bottom row), BOLD signal was extracted at each TR from native space data following preprocessing in AFNI. Specifically, data were slice-time and motion corrected; smoothed; converted to percent signal change relative to the voxel-wise mean for each run; and high-pass filtered. This approach does not rely on the assumptions of the GLM and instead provides a more direct view of condition-related fluctuations in the measured BOLD signal. Since the task contained two jittered ISIs, we extracted event-related BOLD signals from the data at 3 TRs after each task event (to account for the hemodynamic delay) and averaged these values across trials within each condition to obtain an estimate of mean signal for each event. The cue event was defined as TR 1; post-cue delay as TRs 5-6; encoding as TR 7 or 9; delay as TRs 8-9 or 10-11; probe as TR 10, 12, or 14; feedback as TR 11, 13, or 15; and ITI as TRs 12-13, 14-15, or 16-17. The basolateral amygdala (BLA) timecourse revealed that its activity was responsive to value during encoding, and was responsive to cognitive effort during the encoding, delay, and probe periods. The central amygdala (CeA) timecourse revealed that its activity was generally responsive throughout the task periods but did not show differentiation by value or cognitive effort conditions. The nucleus accumbens (NAcc) timecourse revealed that its activity was relatively more responsive to value during the post-cue delay and probe periods, and responsive to cognitive effort during the encoding period. Together, this raw timecourse analysis complements the GLM analysis by providing addition evidence supporting the robustness of the observed value- and cognitive effort-related effects during the task as well as by providing a visualization of activity dynamics in the ROIs throughout each trial.

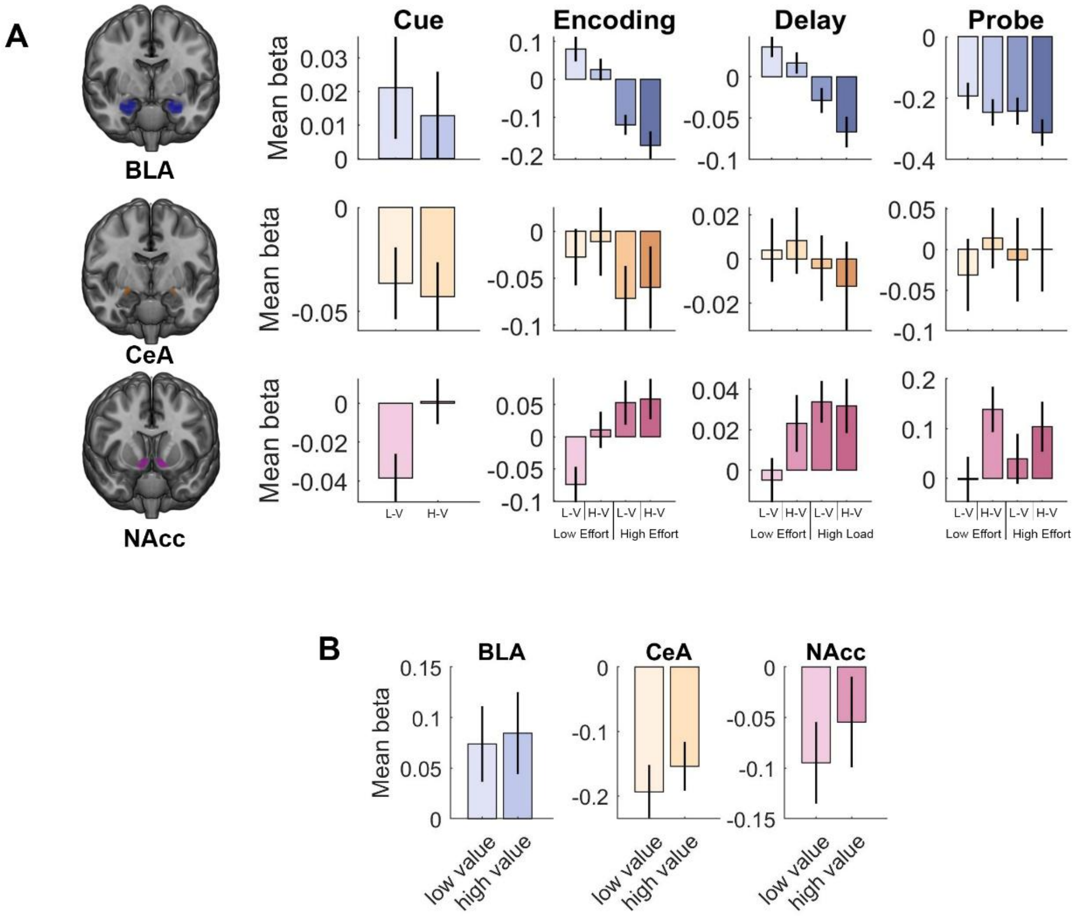

Fig. S3. Control analyses to investigate whether probe value activity in the NAcc is driven by feedback processing. It could be the case the NAcc value coding during probe was driven by value processing during the immediately following feedback period. We examined and ruled out this possibility in two ways. (A) First, we used a variation of GLM1 that included high and low outcome regressors during the feedback period to examine probe-related activity while explicitly controlling for feedback-related value processing. Although probe and feedback regressors of the same value level were moderately correlated (r = .54), this level of collinearity is not excessive and leaves substantial independent variance attributable to the probe period. Notably, the results are highly similar to those reported in the main manuscript—most notably, there is a main effect of value during the probe period for NAcc activity ($F_{1,\,35}$ = 10.55, *p* = .008, Bonferroni corrected). The fact that this effect was robust to explicitly controlling for feedback regressors suggests it reflects genuine probe period value coding. (B) Second, we used a variation of GLM2 that included high and low outcome regressors during the feedback period to examine whether the NAcc (or amygdala) was sensitive to value processing during the feedback period itself. Importantly, in this GLM, value was not modeled at probe (there was only a single probe regressor to capture mean activity). This minimizes probe-feedback collinearity and allows feedback regressors to capture any value-related variance occurring at that time. However, neither NAcc activity (t_{35} = 1.53, *p* = .14, uncorrected) nor amygdala activity (BLA: t < 1; CeA: t_{35} = 1.52, *p* = .14, uncorrected) reflected the effect of value at the time of feedback, even at a liberal uncorrected statistical threshold. These results indicate that feedback-related value processing cannot account for the value signals in NAcc activity observed at probe. *Abbreviations*: L-V, low value; H-V, high-value.

(For completeness, we note that when feedback regressors were added to GLM1, the MVPA analysis no longer found significant value classification during the probe period of low cognitive effort trials based on NAcc activity (p = .19, Bonferroni corrected). This change to non-significance could be due to instability of beta estimates across runs arising from the correlation between probe and feedback regressors. Relative to univariate analyses, MVPA is more sensitive to multicollinearity because it relies on precise beta estimates across voxels and stable beta estimates across runs—requirements that are potentially disrupted by even moderate regressor correlation. This possibility motivated our decision to exclude feedback regressors from the primary (pre-registered) analysis.)

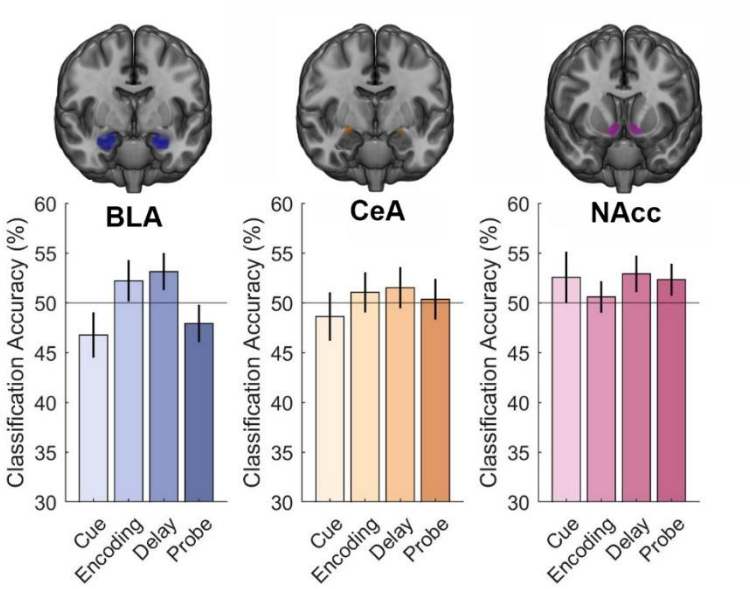

Fig. S4. **Classification of high versus low value (collapsed across cognitive effort)**. Value could not be classified at any time during the task from voxel patterns of any ROIs (all p's Bonferroni corrected): BLA (cue: mean = 46.76%, t_{35} = 1.42, p > .99; encoding: mean = 52.20%, t_{35} = 1.06, p = .45; delay: mean = 53.13%, t_{35} = 1.67, p = .15; probe: mean = 47.92%, t_{35} = 1.11, p > .99), CeA (cue: mean = 48.61%, t_{35} < 1 encoding: mean = 51.04%, t_{35} < 1; delay: mean = 51.50%, t_{35} < 1; probe: mean = 50.35%, t_{35} < 1), NAcc (cue: mean =52.55 %, t_{35} < 1 encoding: mean = 50.58%, t_{35} < 1; delay: mean = 52.89%, t_{35} = 1.58, p =.18; probe: mean = 52.31%, t_{35} = 1.43, p = .24).

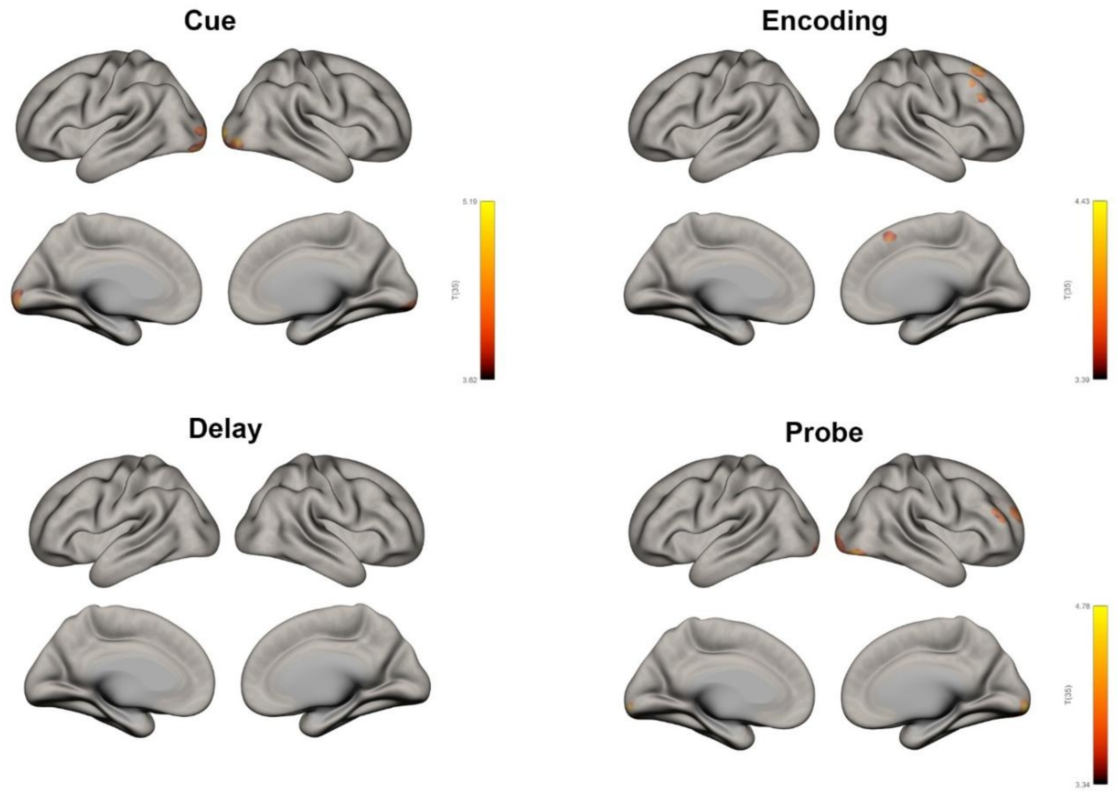

Fig. S5. **Searchlight analysis for the classification of value (collapsed across cognitive effort) during each task event**. During the cue, value could be classified with searchlights that included voxels in the bilateral visual cortex. During encoding, value could be classified from searchlights that included voxels in the lateral PFC and pre-SMA. During the delay, the overall effect of value could not be decoded from any searchlights. During the probe, value could be classified from searchlights that included voxels in the lateral PFC and visual cortex. These maps were thresholded at $Z > 3.1$, $p < .05$ family-wise error rate corrected for cluster extent).

Searchlight Methods: Searchlight analyses do not provide strong tests of anatomical hypotheses, but can offer suggestive evidence that voxels within particular regions may contribute to patterns that discriminate trial types. We defined a sphere with a radius of two voxels (5.8 mm) around each voxel (27 voxels total), which is appropriate for examining small structures including the amygdala and NAcc. The searchlight approach has the benefit of not assuming that all voxels within a region will be informative for the classification. This approach involves a separate SVM for each voxel in the brain using the beta values falling within the searchlight, and assigning the SVM's accuracy to the voxel upon which the searchlight was centered. This generates maps indicating each voxel's decoding accuracy. In line with common practice, the wholebrain searchlight results (accuracy minus chance level performance) were smoothed using a 4 mm FWHM Gaussian kernel, which accounts for differences in localization across participants. Accuracy minus chance maps were entered into a one-sample t-test against 0 in SPM12. We set the threshold for statistical significance at $a = 0.05$ family-wise error (FWE) corrected at the voxel level, small-volume corrected for the combined amygdala and NAcc ROI volume. For wholebrain statistical maps, we used a height thresholded of $p < .001$, and a cluster threshold of $p < .05$ FWE corrected.

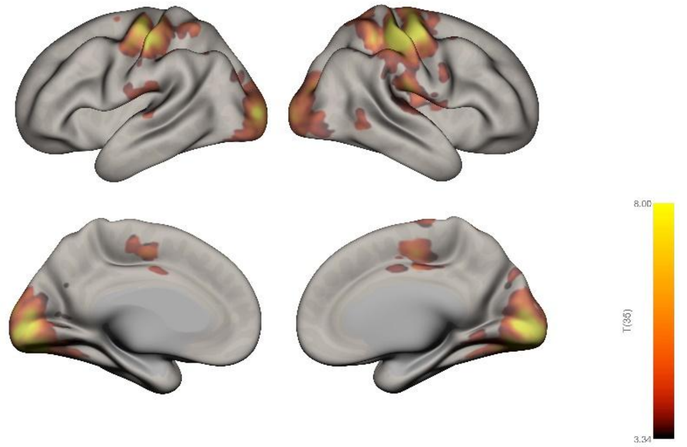

Fig. S6. Searchlight classification analysis for the motor response (left/right button press). Classifiers trained and tested on the left versus right motor response during the probe period revealed significant decoding from searchlights that included somatomotor, posterior insular/opercular, and visual cortical voxels (wholebrain $Z > 3.1$, $p < .05$ family-wise error rate corrected for cluster extent). To examine specificity, we examined whether or not the somatomotor cortex represented value during the probe. We first created spherical ROIs around the group peak coordinates for the left and right somatomotor cortex and then examined whether we could classify high versus low value during the probe using voxel patterns in these ROIs (which were reverse normalized to subjects' native space). This analysis did not include 3 participants due to difficulty warping the group ROIs into their native space. These somatomotor ROIs specifically represented motor response information and not value, as revealed by non-significant accuracy for classifying high versus low value during the probe on low cognitive effort trials (left motor cortex: mean = 49.48%, $t < 1$; right motor cortex: mean = 47.14%, $t_{31} = -1.19$, $p > .99$), and non-significant accuracy for classifying high versus low value during the probe on high cognitive effort trials (left motor cortex: mean = 53.65%, $t_{31} = 1.41$, $p = .17$; right motor cortex: mean = 44.79%, $t_{31} = -2.24$, $p > .99$). In contrast, whereas the BLA and NAcc represented value during the task, they did not represent the motor response, as revealed by non-significant classification of the left/right motor response (BLA: mean = 49.54%, $t < 1$; CeA: mean = 50.69%, $t < 1$; NAcc: mean = 50.93%, $t < 1$). A searchlight analysis revealed a single significant voxel in the NAcc for the motor response classification ($p < .05$, FWE small volume corrected). Thus, value and motor response during the probe are reflected by voxel patterns in distinct sets of regions.

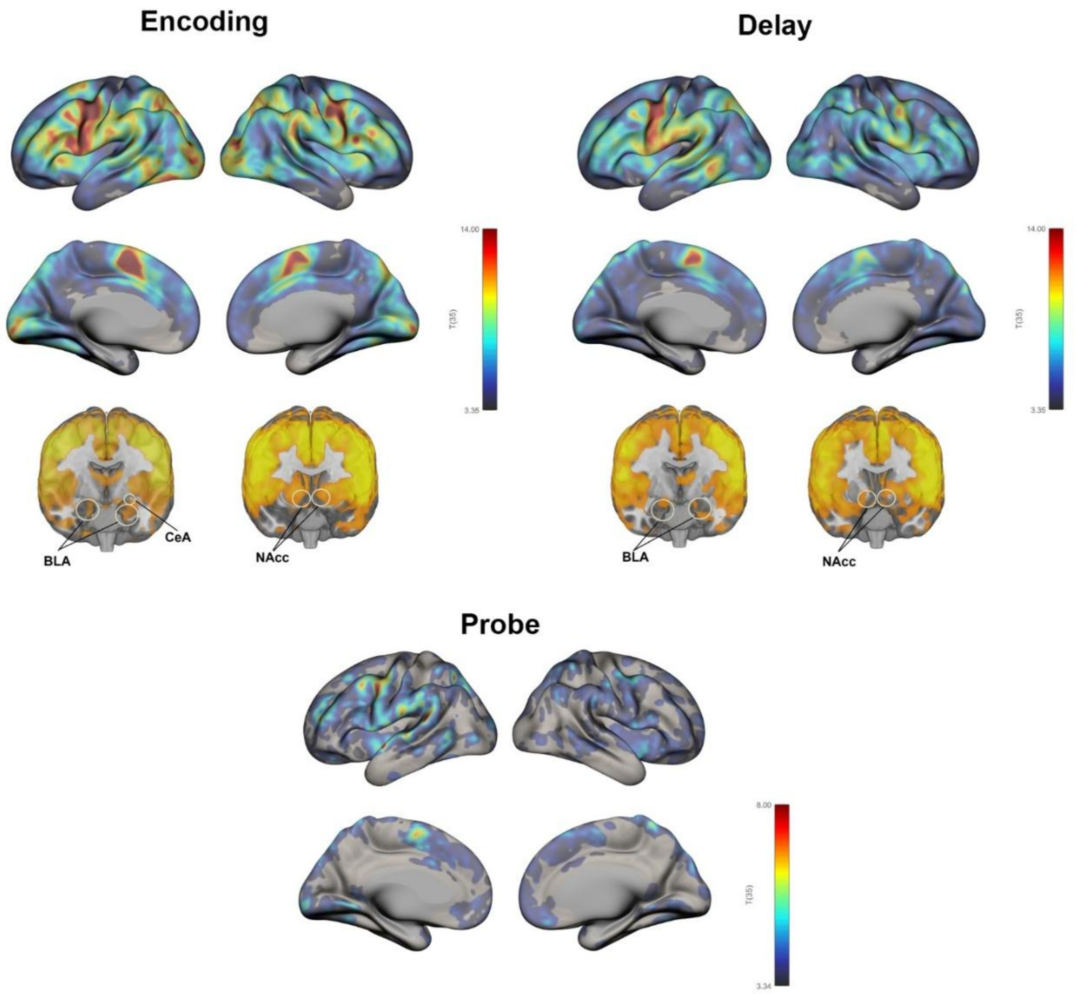

Fig. S7. **Searchlight analysis for the classification of cognitive effort (collapsed across value) during each task event**. During encoding and the delay, cognitive effort could be classified from searchlights that included voxels spanning much of the cortex ($Z > 3.1$, $p < .05$ family-wise error rate corrected for cluster extent) and from searchlights that included voxels in the amygdala and NAcc ($p < .05$, FWE small volume corrected). During the probe, cognitive effort could be classified from searchlights that included voxels in a more select group of cortical regions ($Z > 3.1$, $p < .05$ family-wise error rate corrected for cluster extent).

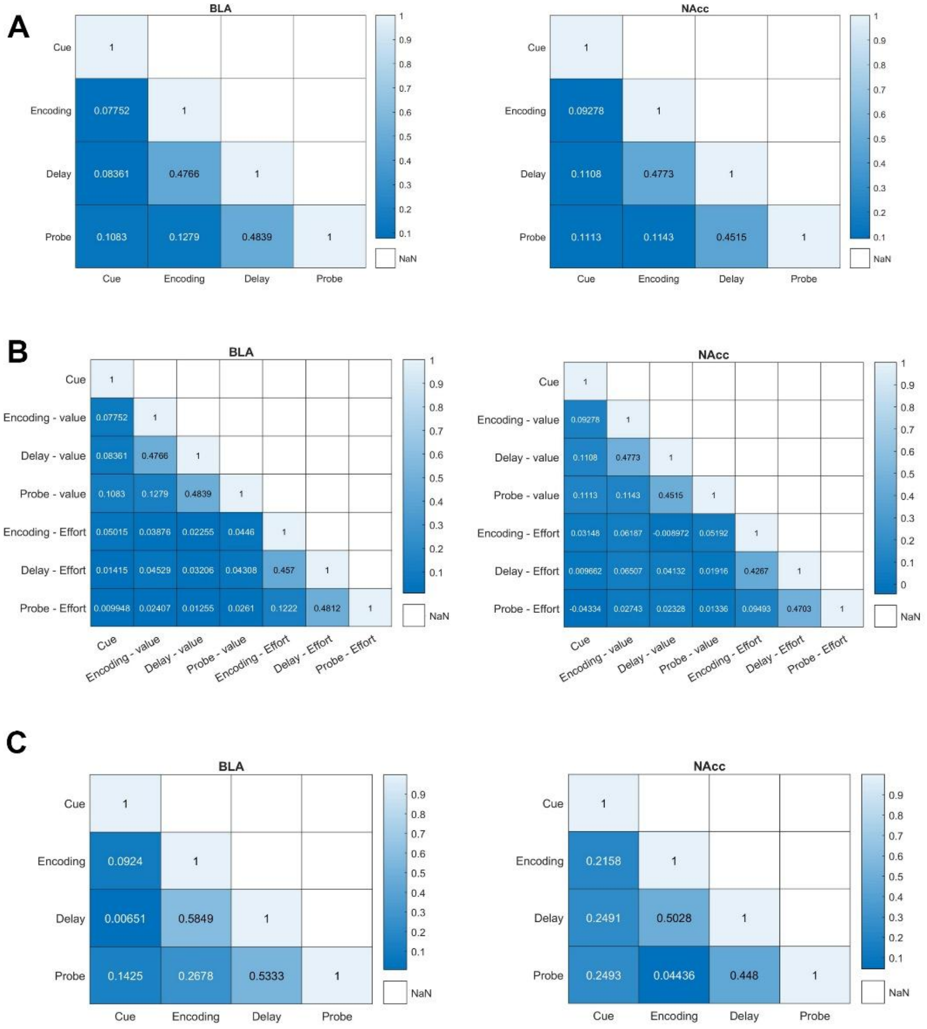

Fig. S8. Pattern similarity analysis results and control analyses. (A) Heatmap values reflect the mean *r* values across participants, indicating similarity of high > low value contrast maps for each pair of task events. There is relatively low similarity between value patterns for the incentive cue and task events, but considerable similarity between value patterns for different task events. **(B)** *Control analysis 1*. A potential concern is that the observed pattern similarity results may reflect the temporal proximity of task events rather than meaningful representational stability. To directly test this possibility, we took advantage of the fact that incentive value and cognitive effort (memory load) were modeled using regressors with identical event timing. Thus, if temporal structure alone drives similarity across events, then substituting incentive value patterns with cognitive effort patterns should produce comparable correlations across task periods. However, this was not the case (see *r* values highlighted with the yellow outline). For both the BLA and NAcc, incentive value patterns during the cue period were not correlated with cognitive effort patterns during any task period (BLA: all mean *r*'s < .051, *p*'s > .05; NAcc: all mean *r* 's < .066, *p*'s > .05). Furthermore, correlations between value and effort patterns within the task (encoding-delay; delay–probe) were non-significant (BLA: all mean *r*'s < .035, *p*'s > .05; NAcc: all mean *r*'s < .06, *p*'s > .05). Because incentive value and cognitive effort regressors share identical temporal structure, these null results indicate that the temporal proximity of events alone does not produce meaningful pattern similarity. **(C)** *Control analysis 2*. A second potential concern is that the task-

related shifts in value representation simply reflected a transition from a lack of value coding during the cue period to the presence of value coding during the task. However, similar results were obtained when only considering the subset of participants who demonstrated above chance-level classification of value during both the cue and task.

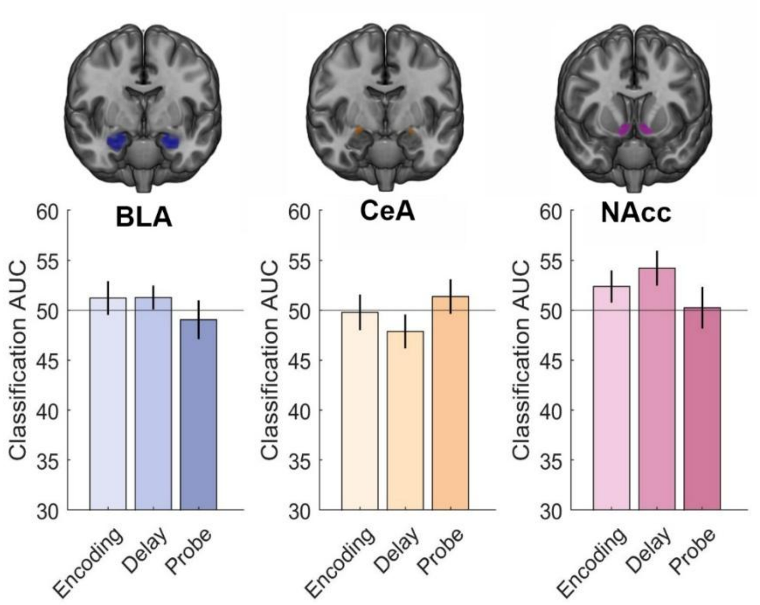

Fig. S9. **Cross-validated cross-classification results**. We tested for the stability of value representations from the cue to the task period by examining whether a classifier trained on the high vs low value distinction during the cue period could accurately discriminate high vs low value during the task periods (or vice versa). If value representations remain relatively stable, we should find above chance classification accuracy. We consider the area under the curve (AUC) in this analysis because it overcomes potential baseline shifts between the different parts of the data that may bias the results. The results indicate that the representation of high vs low value during the cue period is sufficiently distinct from the representation of value during each task period that it is not possible to accurately classify value during the task period based on classifiers trained to classify value during the cue (or vice versa; all *p*'s > .05, Bonferroni corrected; note that the NAcc delay effect was marginal at $p = .064$, Bonferroni corrected). In sum, these findings support the conclusion that the value representation evolves as the cognitive context is introduced.

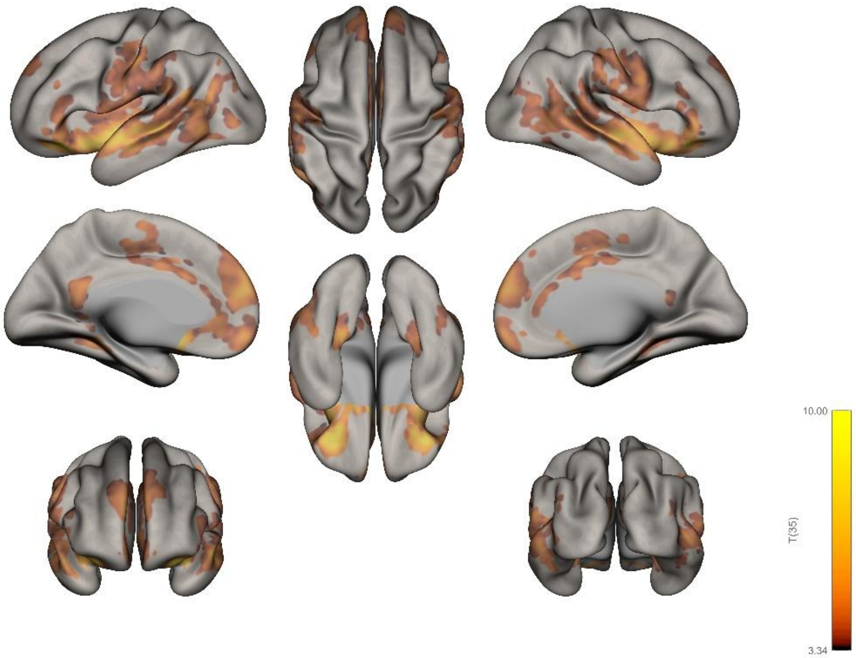

Fig. S10. **Background functional coupling for the CeA.** The CeA was significantly coupled with voxels located within the DLPFC ROI (*p* < .05 FWE small-volume corrected), with peaks in the right inferior frontal sulcus (x = 46, y = 19, z = 26; x = 46, y = 27, z = 20) and left inferior frontal gyrus (x = -46, y = 21, z = 20), and was coupled with voxels located within the frontoparietal working memory ROI (*p* < .05 FWE small-volume corrected), with a peak in the left anterior insula (x = -26, y = 19, z = -3).

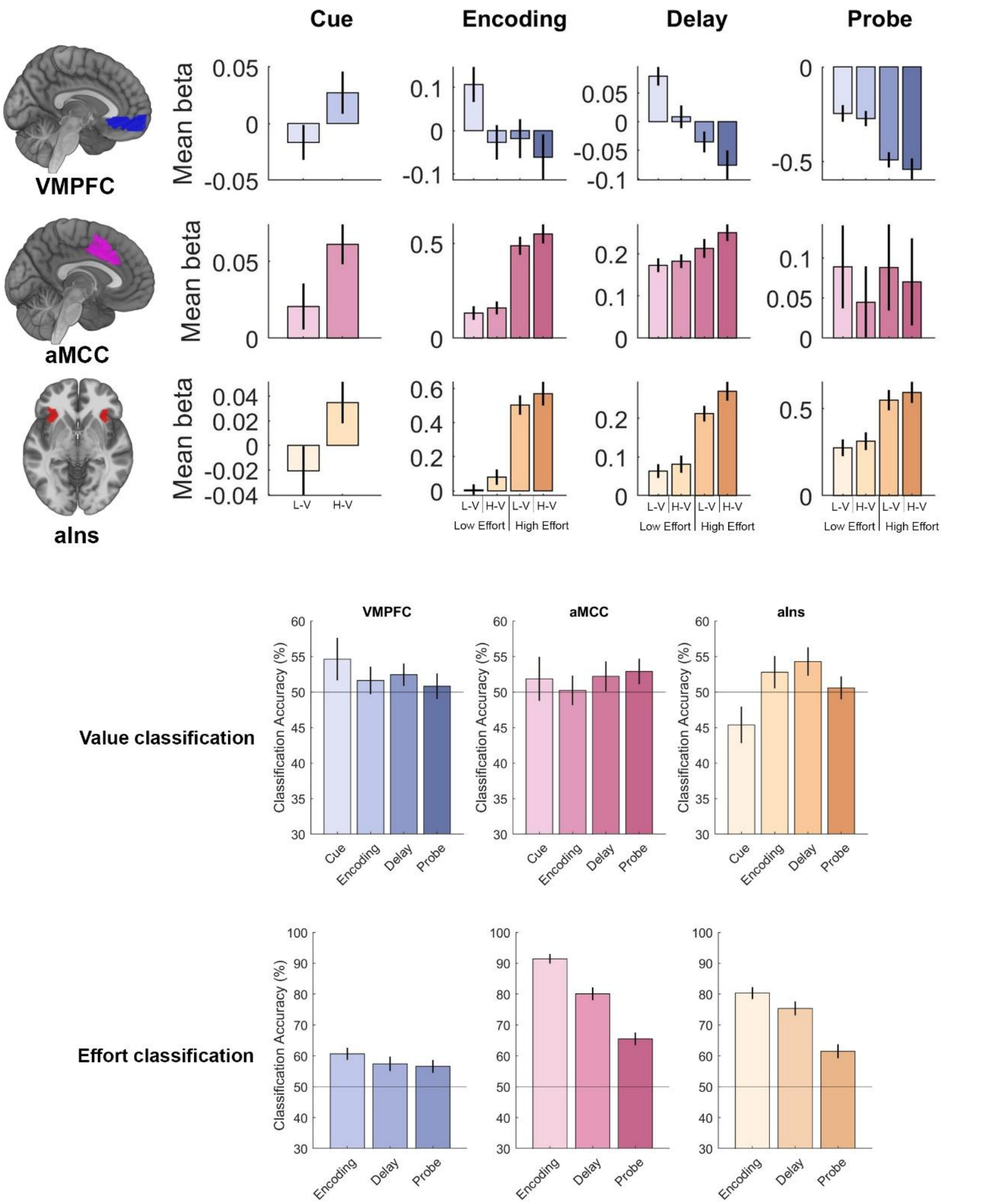

Fig. S11. **Exploratory analysis of cortical ROIs**. ROIs were derived from the Schaefer 400 parcel 7-network atlas. The ventromedial prefrontal cortex (VMPFC) was defined as parcels 168, 169 and 379 (mean = 255.94 voxels, SD = 31.14 voxels). Following Yee et al., 2021 the anterior mid-cingulate cortex (aMCC; also known as dorsal ACC) was defined as parcels 107, 108, 110, 311, 312 (mean = 359.94 voxels, SD = 42.45 voxels). The anterior insula (aIns) was defined as parcels 99, 101, 143, 306, 340 (mean = 221.08 voxels, SD = 24.70 voxels). **Univariate results**. VMPFC BOLD signal did not reflect value during the cue (t_{35} = 1.93, p = .19), but did show a main effect of value during the encoding period ($F_{1, 35}$ = 10.01, p = .01) and delay period ($F_{1, 35}$ = 14.31, p = .002), but not during the probe period ($F_{1, 35}$ = 1.70, p = .60). VMPFC BOLD signal reflected

cognitive effort during the delay ($F_{1, 35}$ =24.95, $p < .001$) and probe ($F_{1, 35}$ =49.30, $p < .001$), but not during encoding ($F_{1, 35}$ = 3.73, p = .18). aMCC BOLD signal reflected value during the cue (t_{35} = 2.68, p = .034), but not during the other task periods (all p's > .12). aMCC BOLD signal reflected cognitive effort during the encoding period ($F_{1, 35}$ = 65.43, $p < .001$) and delay period ($F_{1, 35}$ = 9.75, p = .01), but not during probe ($F < 1$). AIns BOLD signal reflected value during the cue (t_{35} = 3.27, p = .007), and delay period ($F_{1, 35}$ = 6.64, p = .043), but not during the encoding period ($F_{1, 35}$ = 3.15, p = .25) or probe period ($F_{1, 35}$ = 1.91, p = .53). aINs BOLD signal reflected cognitive effort during all task periods (all p's < .001). There were no significant interactions. **MVPA results**. MVPA demonstrated that cognitive effort could be classified from the voxel patterns of each cortical ROI during each task period (all p's < .05), whereas value could not be classified during any task period (all p's > .05). Furthermore, value could not be classified when separately examining high and low cognitive effort trials (not shown; all p's > .05). Note that all p-values are Bonferroni corrected. Abbreviations: L-V, low value; H-V, high-value.

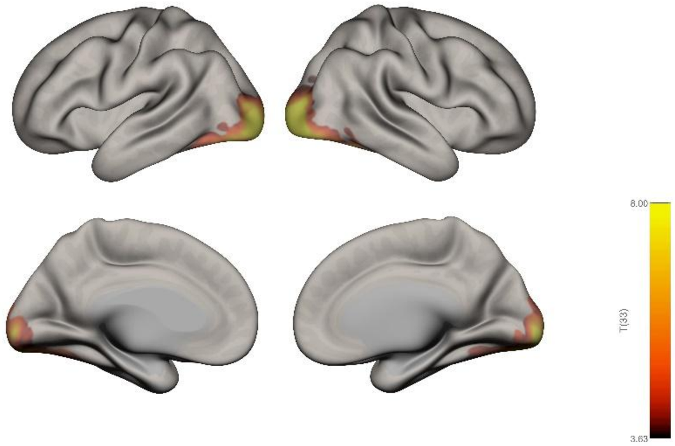

Fig. S12. **Searchlight analysis for the classification of cue type based on visual features (money bags versus lines)**. Cue visual features were orthogonal to cue value and were modeled in a variant of GLM 2 which included four cue regressors (dollar amount – high value, dollar amount – low value, horizontal lines – high value, horizontal lines – low value). We then conducted a searchlight analysis to decode the effect of cue type (dollar amount versus horizontal lines), thus isolating the visual features of the cue independently of their predicted value. We found that visual features could be classified from searchlights that included voxels in the bilateral occipital pole, lateral occipital cortex, lingual gyrus, fusiform gyrus, inferior temporal gyrus, and cerebellum crus 1 (wholebrain $Z > 3.1$, $p < .05$ family-wise error rate corrected for cluster extent). No significant voxels were located in the amygdala or NAcc. ROI-based analyses also revealed non-significant classification of of cue visual features using voxel patterns from the amygdala (BLA: mean = 50.37%, $t_{35} < 1$; CeA: mean = 48.65%, $t_{35} < 1$) or NAcc (mean = 49.51%, $t_{35} < 1$). Thus, neither the amygdala nor NAcc are sensitive to cue visual features, independent of value.

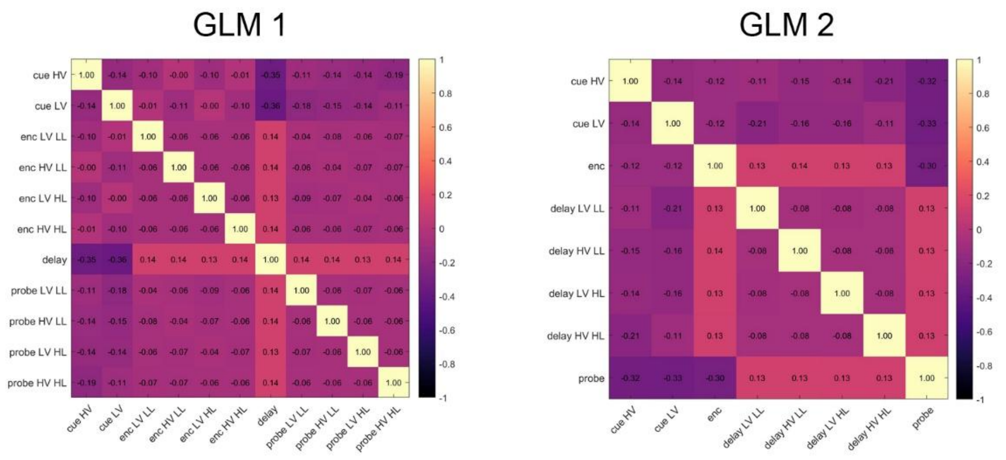

Fig. S13. Full correlation matrix of task regressors. With working memory tasks that have several sequential task events, there is the issue of dependencies between those events (in our case, cue, encoding, delay, and probe periods). The crucial issue for analysis and interpretation concerns dependencies between the task regressors modeling the experimental conditions of interest rather than the temporal proximity of task events *per se*. We used two approaches to minimize collinearity among sequential task periods/conditions. First, the task was carefully designed with jittered intervals (4 - 8 sec post-cue and 4 - 8 sec delay length), which effectively decorrelated cue, encoding, and delay periods within the design matrix. Second, although the probe period (2 sec) always immediately followed the delay period, we minimized collinearity between condition-specific regressors by adopting a selective modeling approach (as used in prior working memory studies). In this approach, the conditions of interest (value x cognitive effort) were modeled for one event (e.g., delay), but were collapsed across for the adjacent event (e.g., probe) in the GLM, and vice versa in a complementary model. In GLM1, condition x cognitive effort was not modeled for the delay, whereas in GLM2 condition x cognitive effort was not modeled for encoding or probe. This approach significantly reduces collinearity between the regressors of interest across sequential periods. The empirical correlations between all pairs of task regressors were all low (all $r \leq .14$). Abbreviations: low value, LV; high value HV; low value low load, LV LL, high value low load, HV LL, low value high load, LV HL, high value high load, HV HL.

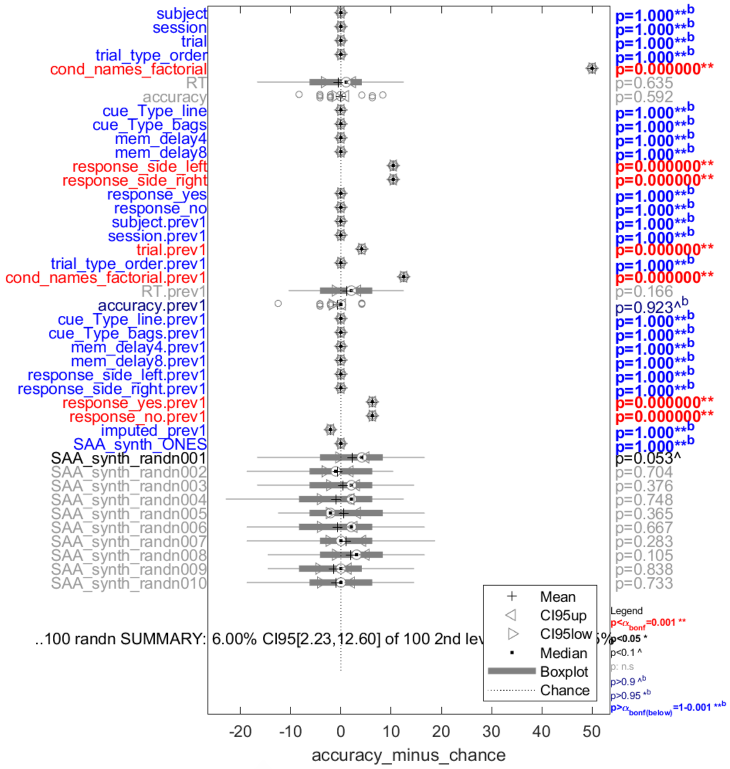

Fig. S14. **Same Analysis Approach (SAA) results for the classification of value (during high cognitive effort trials) analysis**. The SAA (1) is a rigorous method for determining whether any extraneous features of the task design could, in theory, bias classification accuracy for the key variables of interest. For each of analysis, this approach examines any task-related variables specified by the user, as well as the same variables shifted by one trial to examine trial history effects (the prev1 variables), and also includes simulated null data sets (N = 100) to examine the false positive rate. In the primary analysis, we found that value could be classified from the voxel pattern of the BLA during the delay period of high cognitive effort trials. The key question is whether any extraneous task variables flagged as significant by the SAA could potentially account for this finding. We address these cases below.

There was a significant effect of response side (response left/response right), with a mean classification of 10.42% above chance. Although response side and value condition were counterbalanced across participants, there was a slight within-person imbalance (e.g., N = 7 high value trials paired with a left response and N = 12 low value trials paired with a left response). This raises the possibility that classifier performance could reflect not only high versus low value, but potentially also response side information. However, in our primary analyses, none of the ROIs showed significant classification of value during the probe period of high cognitive effort trials—the only task period in which a left or right response was actually made. Thus, the imbalance is not a concern for interpreting delay-related classification, as no motor response occurred during this period. It is also worth noting that response side could be classified from voxel patterns in somatomotor regions but not from amygdala or NAcc voxel patterns (see **Fig. S6** for ROI and searchlight results).

There was a significant effect of previous trial number, with a mean classification of 4.17% above chance. This was due to the fact that across all runs, trial n was more likely to precede one of the conditions (e.g., trial 1 was more likely to precede a low value trial on trial 2 (N = 2) than precede

a high value trial on trial 2 (N = 1). There was a difference in count of 1 for all trials. It seems highly unlikely that participants were even implicitly tracking whether a low value or high value condition occurred on a given trial number (e.g., trial 2) and were sensitive to whether one condition was preceded by a particular trial number once or twice across the entire experiment. At most, this small imbalance could bias classifier performance during the cue period, but given the lack of any plausible mechanism for participants to process this aspect of the task structure and given the lack of significant classification of value during the cue, this is not an issue.

There was a significant effect of previous trial type, with a mean classification of 12.5% above chance. For context, let us first consider the four trial types of the factorial design (value x cognitive effort). Given the number of trials per run, and the constraint of no more than two trials in a row of the same condition, it is not possible to balance all previous trial effects. We balanced the number of times that a given condition was preceded by each of the other three conditions, but there were fewer repeats of the same trial type. This was done intentionally to make the task psychologically unpredictable and interesting. Thus, while it is theoretically possible that participants could expect a switch in condition to occur, they could not anticipate which condition of the factorial design would be next, given that a switch to any of the other three conditions was equally likely to occur. We confirmed this by creating a variable reflecting just these switch trials and found that the previous trial effect was reduced to 3% below chance classification accuracy. Now, let us consider just the effect of value—the focus of this analysis. Across all trials, there were more switches (N = 54 from low to high value or vice versa) than repeats of the same value condition (N = 36). This raises the that, in addition to the distinction between high and low value, classifier performance could potentially also reflect expectation/prediction errors. If the expectation is that there will be a switch in trial type (because this happens more often), then the transition from a high value trial to another high value trial could create a positive prediction error (it is better than expected), and the transition from a low value trial to another low value trial could create a negative prediction error (it is worse than expected), and a switch trial in either direction would align with expectations and create minimal prediction error. Notably, this potential influence of expectation would, in theory, exaggerate the difference between high and low value trials, especially during the cue period when the current trial type is revealed and expectations are confirmed or violated. However, value could not be classified from any ROI voxel patterns during the cue, suggesting that expectancy/prediction error–related effects are unlikely to have influenced classifier performance. Therefore, previous trial type effects do not appear to account for our main findings of delay period encoding of value by the BLA.

There was also a significant effect of previous response scale (yes response/no response), with a mean accuracy of 6.25% above chance. There was a slight imbalance within participants (e.g., N = 13 yes response on the previous trial and a high value condition on the current trial, versus N = 10 yes response on the previous trial and a low value condition on the current trial). If the contingency between the previous yes/no response and current trial condition were to affect classifier performance, it would be most likely to occur during the cue period when the current value condition is revealed. However, as noted, there was no significant classification of value during the cue period and there is no a priori reason to expect that the slight imbalance in the previous yes/no response-current condition mapping would selectively influence classifier performance during the delay period of high cognitive effort trials and just for the BLA and not other ROIs. Thus, the previous yes/no response effect is unlikely to account for our main findings of delay period encoding of value by the BLA.

The analysis also tested for any potential systematic bias that may arise from the first trial of each run if the design is not fully randomized. We can do this by testing whether the condition of the first trial can be predicted from what happened on the previous trial. Because the very first trial of a run has no true “previous trial” we used a simple imputation method: we recycled the last trial of the run and treated it as if it were the trial that came before the first one. This choice is pragmatic—the last trial is a real trial with a plausible condition type, but should be unrelated to the first trial if the design is fully randomized. To test for this, we created an indicator variable (imputed_prev1) that is a 1 for all imputed data and 0 otherwise. If classification is significant, it

means there is the potential for the first trial to bias classification accuracy due to the design not being fully randomized. The analysis revealed a significant effect for the imputed_prev1 variable (mean accuracy of 2.08% below chance). However, because this effect is below chance, it would only potentially suppress small effects of interest and is not a concern for creating false positive results by inflating classification accuracies.

The analysis also included simulated null data sets that do not contain the experimental effect of interest, but come from a generative model that captures as many aspects of the real data as possible (distribution, autocorrelation, effect size, variation across measurements, trials, runs, and subjects). By applying the same analysis to multiple versions of simulated null data, we can test if the false positive rate (the alpha level) is at the expected (5%) level. We generated 100 simulated null data sets and found a mean false positive rate of 6% with a 95%-CI of [2.23%-12.6%], which is perfectly in line with the assumed true underlying false positive rate of 5%.

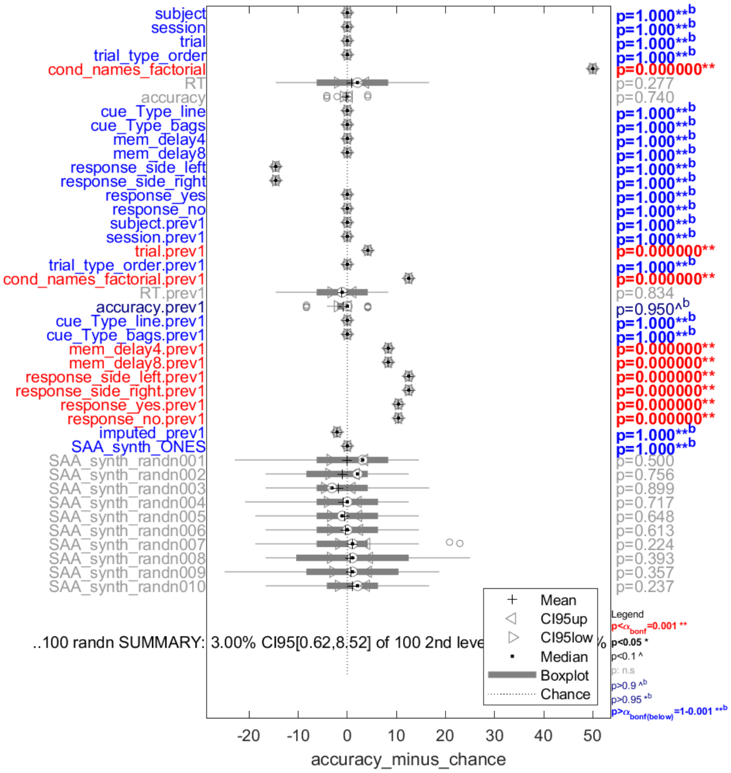

Fig. S15. **Same Analysis Approach (SAA) results for the classification of value (during low cognitive effort trials) analysis.** The SAA (1) is a rigorous method for determining whether any extraneous features of the task design could, in theory, bias classification accuracy for the key variables of interest. For each of analysis, this approach examines any task-related variables specified by the user, as well as the same variables shifted by one trial to examine trial history effects (the prev1 variables), and also includes simulated null data sets (N = 100) to examine the false positive rate. In the primary analysis, we found that value could be classified from the voxel patterns of the BLA during the encoding period of low cognitive effort trials and from the voxel patterns of the NAcc during the probe period of low cognitive effort trials. The key question is whether any extraneous task variables flagged as significant by the SAA could potentially account for these findings. We address these cases below.

There was a significant effect of response side (left/right), with a mean classification of 14.58% below chance. Although response side and value condition were counterbalanced across participants, there was a slight within-person imbalance (e.g., N = 15 high value trials paired with a left response and N = 14 low value trials paired with a left response). This raises the possibility

that classifier performance during the probe could reflect not only high versus low value, but potentially also response side information. Thus, while this is unlikely to bear on the significant classification of value from BLA voxel patterns during the encoding period, it could in theory affect classifier performance for decoding value during the probe period from the NAcc voxel pattern. To rule out this possibility, we ran a subset analysis. The subset analysis involved excluding (scrubbing) a random subset of trials so that each response (left or right) was paired equally often with both conditions. We then re-ran the analyses. This procedure was repeated twice to ensure that the results were robust and not driven by the specific subset of trials that were excluded. These subset analyses revealed that value could still be classified from NAcc voxel patterns (variation 1: mean = 56.71%, t_{35} = 3.29, p = .001; variation 2: mean = 56.71%, t_{35} = 2.47, p = .009), indicating that the slight bias in response side – condition mapping cannot account for the significant classification of value from NAcc voxel patterns.

There was a significant effect of previous trial number, with a mean classification of 4.17% above chance. This was due to the fact that across all runs, trial n was more likely to precede one of the conditions (e.g., trial 1 was more likely to precede a low value trial on trial 2 (N = 2) than precede a high value trial on trial 2 (N = 1). There was a difference in count of 1 for all trials. It seems highly unlikely that participants were even implicitly tracking whether a low value or high value condition occurred on a given trial number (e.g., trial 2) and were sensitive to whether one condition was preceded by a particular trial number once or twice across the entire experiment. At most, this small imbalance could bias classifier performance during the cue period, but given the lack of any plausible mechanism for participants to process this aspect of the task structure and given the lack of significant classification of value during the cue, this is not an issue.

There was a significant effect of previous trial type, with a mean classification of 12.5% above chance. As noted for the previous analysis, we balanced the number of times that a given condition was preceded by each of the other three conditions, and when we consider just these switch trials, the previous trial effect was reduced to 0% above chance classification accuracy. And again, if any expectation/prediction error effects arise due to the fact that the value condition switches more often than it repeats, this would be expected to primarily impact the cue period when the current value condition is revealed, yet we did not find significant classification of value during the cue period for any ROI. It is unlikely that previous trial type effects could account for our main findings of classification of value from encoding period BLA patterns and classification of value from probe period NAcc patterns.

There was also a significant effect of previous memory delay length, with a mean of 8.33% above chance. There was a slight imbalance within participants (e.g., N = 11 8-sec delay on the previous trial and a high value condition on the current trial, versus N = 10 8-sec delay on the previous trial and a low value condition on the current trial). Although it seems unlikely that participants would register this very weak contingency between the previous delay length and current trial condition, if this were to occur and potentially influence classifier performance, it would be most likely to have an effect during the cue period when the current trial condition is revealed. But as noted above, we did not find significant classification during the cue for any ROI, making it unlikely that the memory delay length history impacted our main findings.

There was a significant effect of previous response side (left/right), with a mean classification of 12.5% above chance. This was due to a slight within-participant imbalance in the mapping between previous response side and current trial condition (e.g., N = 13 left response on the previous trial followed by a high value trial and N = 8 trials with a left response on the previous trial followed by a low value trial). If participants registered this imbalance—either implicitly or explicitly—it could, in theory, bias classification accuracy during the current trial, especially during the cue period when the current trial value (high or low) condition is first revealed. That is, classifier performance during the cue could reflect not just high versus low value, but potentially also prior response history. However, we did not find significant classification of value during the cue period from the voxel pattern of any ROI, suggesting that previous response information did not influence classifier performance during that period. It seems highly unlikely that previous

response side could selectively influence classification of value only during later periods—during the encoding period for the BLA and only during the probe period for the NAcc. Thus, the imbalance in prior response history is unlikely to account for the findings reported in the main manuscript.

There was also a significant effect of previous response scale (yes/no response), with a mean accuracy of 10.42% above chance. There was a slight imbalance within participants (e.g., N = 13 yes response on the previous trial and a high value condition on the current trial, versus N = 10 yes response on the previous trial and a low value condition on the current trial). If the contingency between the previous yes/no response and current trial condition were to affect classifier performance, it would be most likely to occur during the cue period when the current value condition is revealed. However, as noted, there was no significant classification of value during the cue period and there is no *a priori* reason to expect that the slight imbalance in the previous yes/no response-current condition mapping would selectively influence classifier performance during later task periods. Thus, the previous yes/no response effect is unlikely to account for our main findings.

The analysis also tested for any potential systematic bias that may arise from the first trial of each run if the design is not fully randomized. We can do this by testing whether the condition of the first trial can be predicted from what happened on the previous trial. Because the very first trial of a run has no true “previous trial” we used a simple imputation method: we recycled the last trial of the run and treated it as if it were the trial that came before the first one. This choice is pragmatic—the last trial is a real trial with a plausible condition type, but should be unrelated to the first trial if the design is fully randomized. To test for this, we created an indicator variable (imputed_prev1) that is a 1 for all imputed data and 0 otherwise. If classification is significant, it means there is the potential for the first trial to bias classification accuracy due to the design not being fully randomized. The analysis revealed a significant effect for the imputed_prev1 variable (mean accuracy of 2.08% below chance). However, because this effect is below chance, it would only potentially suppress small effects of interest and is not a concern for creating false positive results by inflating classification accuracies.

The analysis also included simulated null data sets that do not contain the experimental effect of interest, but come from a generative model that captures as many aspects of the real data as possible (distribution, autocorrelation, effect size, variation across measurements, trials, runs, and subjects). By applying the same analysis to multiple versions of simulated null data, we can test if the false positive rate (the alpha level) is at the expected (5%) level. We generated 100 simulated null data sets and found a mean false positive rate of 3%, with a 95%-CI of [0.62%-8.52%], which is perfectly in line with the assumed true underlying false positive rate of 5%.

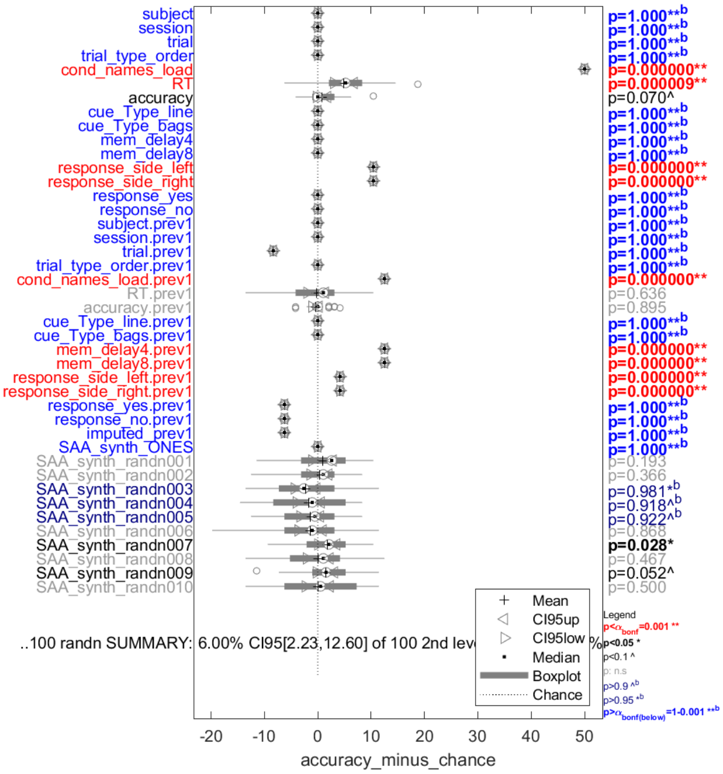

Fig. S16. Same Analysis Approach (SAA) results for the classification of cognitive effort (collapsed across value) analysis. The SAA (1) is a rigorous method for determining whether any extraneous features of the task design could, in theory, bias classification accuracy for the key variables of interest(1). For each of analysis, this approach examines any task-related variables specified by the user, as well as the same variables shifted by one trial to examine trial history effects (the prev1 variables), and also includes simulated null data sets (N = 100) to examine the false positive rate. In the primary analysis, we found that cognitive effort could be classified from the voxel patterns of the BLA during the encoding period. The key question is whether any extraneous task variables flagged as significant by the SAA could potentially account for this finding. We address these cases below.

There was a significant effect of RT, with a mean of 4.90% above chance, which reflects the expected behavioral enhancement for low cognitive effort versus high cognitive effort trials. Given that this behavioral effect is tied to the experimental manipulation of interest, and predicted to arise from the neural patterns described in the main text, it is not a concern for our purposes.

There was a significant effect of response side (left response/right response), with a mean classification of 10.42% above chance. Although response side and cognitive effort condition were counterbalanced across participants, there was a slight within-person imbalance (e.g., N = 29 low cognitive effort trials paired with a left response, versus N = 19 high cognitive effort trials paired with a left response). This raises the possibility that classifier performance could reflect not only high versus low cognitive effort, but potentially also the motor response. However, in our primary analyses, none of the ROIs showed significant classification of cognitive effort during the probe period—the only task period in which a left or right response was actually made. Thus, the imbalance is not a concern for interpreting the main finding of cognitive effort-related classification during the encoding period, as no motor response occurred during this period. Moreover, as noted earlier, response side could be classified from voxel patterns in somatomotor regions but not from amygdala or NAcc voxel patterns (see **Fig. S6** for ROI and searchlight results).

There was a significant effect of previous trial number, with a mean of 8.33% below chance. This was due to the fact that across all runs, trial n was more likely to precede one of the conditions (e.g., trial 1 was more likely to precede a low cognitive effort trial on trial 2 (N = 2) than precede a high cognitive effort trial on trial 2 (N = 1). The classification accuracy was likely higher than for the previous analyses because in this case we are considering the overall effect of cognitive effort, meaning high versus low cognitive effort on both low value and high value trials. Just as in the previous analyses, there was a difference in count of 1 for all trials. Again, it seems highly unlikely that participants were even implicitly aware of whether a low cognitive effort or high cognitive effort condition occurred on a given trial number (e.g., trial 2) and were sensitive to whether one condition was preceded by a particular trial number once or twice across the entire experiment. It is highly unlikely that this small imbalance was detected by participants and able to carry over all the way until the encoding period when the cognitive effort condition was revealed, and is therefore not an issue.

There was also a significant effect of previous trial type, with a mean of 12.5% above chance. As noted above, when we consider the factorial conditions and just the switch trials, the previous trial type effect disappears (0% above chance). When we consider just cognitive effort —the focus of this analysis—there were more switch trials (N = 58; from low to high cognitive effort or vice versa) than repeat trials (N = 32). This leaves open the possibility of expectation effects related to cognitive effort. Specifically, if participants came to expect a switch in cognitive effort on each trial, then repeat trials could evoke effort-related prediction errors: a high cognitive effort to high cognitive effort transition could be perceived as unexpectedly demanding (a “negative” prediction error), while a low cognitive effort to low cognitive effort transition could be perceived as unexpectedly easy (a “positive” prediction error). Switch trials would align with expectations and elicit minimal prediction error. Thus, classifier performance, could in theory, reflect both the distinction between high and low cognitive effort and the influence of these expectation/prediction error effects, especially during the encoding period when the current cognitive effort condition is revealed. Crucially, this is not a problem for our purposes, because we are not interested in whether the amygdala and NAcc represent working memory cognitive effort *per se* (i.e., whether they contribute to the content of working memory). Rather, we are interested in whether they represent the moment-to-moment experience of subjective effort, which we think is part of determining the value of performing the task. Crucially, momentary estimates of subjective effort likely depend on both the actual task condition and any mismatch between expected and actual effort demands. From this perspective, expectation/prediction error effects are not a confound, but a meaningful part of the neural representation we are investigating.

There was also a significant effect of previous memory delay length (4-sec/8-sec), with a mean of 12.5% above chance. There was a slight imbalance within participants (e.g., N = 28 8-sec delay on the previous trial and a high cognitive effort condition on the current trial, versus N = 18 8-sec delay on the previous trial and a low cognitive effort condition on the current trial). It seems unlikely that participants would register a contingency between the previous delay length and current cognitive effort condition given that they are separated by multiple intervening events (probe, feedback, inter-trial interval, incentive cue, inter-stimulus interval). However, if it did influence classifier performance, this would be most likely to occur during the encoding period when the current cognitive effort condition is first revealed. We would expect this potential influence to affect classifier performance for all ROIs, yet we only found a significant effect for the BLA. Nevertheless, we ran a subset analysis to ensure that BLA classification did not reflect this systematic feature of our task design. The subset analysis involved excluding (scrubbing) a random subset of trials so that each previous delay length (4 secs or 8 secs) was paired equally often with both conditions. This analysis simultaneously involved balancing the number of previous responses (left or right) that were paired with each condition, because as discussed below, previous response also showed a significant effect in the SAA analysis and could potentially bias BLA classification of cognitive effort during encoding. We then re-ran the analyses with the subset of trials excluded, and this procedure was repeated twice to ensure that the results were robust and not driven by the specific subset of trials that were excluded. These subset analyses revealed that cognitive effort could still be classified from BLA voxel patterns

(variation 1: mean = 55.67%, t_{35} = 3.03, p = .002; variation 2: mean = 53.36%, t_{35} = 1.84, p = .037), indicating that the slight bias in the mapping between previous delay length (and between previous response) and condition did not influence the results.

There was a significant effect of previous response side (left/right), with a mean classification of 4.17% above chance. This was due to a slight within-participant imbalance in the mapping between previous response side and current trial condition (e.g., N = 25 left response on the previous trial followed by a high cognitive effort trial and N = 21 trials with a left response on the previous trial followed by a low cognitive effort trial). It seems unlikely that participants would register a contingency between the previous response and current cognitive effort condition given that they are separated by multiple intervening events (feedback, inter-trial interval, incentive cue, inter-stimulus interval). However, if it did influence classifier performance, this would be most likely to occur during the encoding period when the current cognitive effort condition is first revealed. We would expect this potential influence to affect classifier performance for all ROIs, yet we only found a significant effect for the BLA. Nevertheless, we ran a subset analysis as described above and found that this bias in the task design could not account for the BLA classification results.

The analysis also tested for any potential systematic bias that may arise from the first trial of each run if the design is not fully randomized. We can do this by testing whether the condition of the first trial can be predicted from what happened on the previous trial. Because the very first trial of a run has no true “previous trial” we used a simple imputation method: we recycled the last trial of the run and treated it as if it were the trial that came before the first one. This choice is pragmatic—the last trial is a real trial with a plausible condition type, but should be unrelated to the first trial if the design is fully randomized. To test for this, we created an indicator variable (imputed_prev1) that is a 1 for all imputed data and 0 otherwise. If classification is significant, it means there is the potential for the first trial to bias classification accuracy due to the design not being fully randomized. The analysis revealed a significant effect for the imputed_prev1 variable (mean accuracy of 6.25% below chance). However, because this effect is below chance, it would only potentially suppress small effects of interest and is not a concern for creating false positive results by inflating classification accuracies.

The analysis also included simulated null data sets that do not contain the experimental effect of interest, but come from a generative model that captures as many aspects of the real data as possible (distribution, autocorrelation, effect size, variation across measurements, trials, runs, and subjects). By applying the same analysis to multiple versions of simulated null data, we can test if the false positive rate (the alpha level) is at the expected (5%) level. We generated 100 simulated null data sets and found a mean false positive rate of 6% with a 95%-CI of [2.23%-12.6%], which is perfectly in line with the assumed true underlying false positive rate of 5%.
